# Prognostic value of dobutamine stress myocardial perfusion echocardiography in patients with known or suspected coronary artery disease and normal left ventricular function

**DOI:** 10.1371/journal.pone.0172280

**Published:** 2017-02-24

**Authors:** Angele A. A. Mattoso, Jeane M. Tsutsui, Ingrid Kowatsch, Vitória Y. L. Cruz, João C. N. Sbano, Henrique B. Ribeiro, Roberto Kalil Filho, Thomas R. Porter, Wilson Mathias

**Affiliations:** 1 Heart Institute (InCor), University of São Paulo Medical School, São Paulo, Brazil; 2 University of Nebraska Medical Center, Omaha, United States of America; Universitatsklinikum Wurzburg, GERMANY

## Abstract

**Objective:**

We sought to determine the prognostic value of qualitative and quantitative analysis obtained by real-time myocardial perfusion echocardiography (RTMPE) in patients with known or suspected coronary artery disease (CAD).

**Background:**

Quantification of myocardial blood flow reserve (MBFR) in patients with CAD using RTMPE has been demonstrated to further improve accuracy over the analysis of wall motion (WM) and qualitative analysis of myocardial perfusion (QMP).

**Methods:**

From March 2003 to December 2008, we prospectively studied 168 patients with normal left ventricular function (LVF) who underwent dobutamine stress RTMPE. The replenishment velocity reserve (β) and MBFR were derived from RTMPE. Acute coronary events were: cardiac death, myocardial infarction and unstable angina with need for urgent coronary revascularization.

**Results:**

During a median follow-up of 34 months (5 days to 6.9 years), 17 acute coronary events occurred. Abnormal β reserve in ≥2 coronary territories was the only independent predictor of events hazard ratio (HR) = 21, 95% CI = 4.5–99; p<0.001). Both, abnormal β reserve and MBFR added significant incremental value in predicting events over qualitative analysis of WM and MP (χ2 = 6.6 and χ2 = 24.6, respectively; p = 0.001 and χ2 = 6.6 and χ2 = 15.5, respectively; p = 0.012, respectively). When coronary angiographic data was added to the multivariate analysis model, β reserve remained the only predictor of events with HR of 21.0 (95% CI = 4.5–99); p<0.001.

**Conclusion:**

Quantitative dobutamine stress RTMPE provides incremental prognostic information over clinical variables, qualitative analysis of WM and MP, and coronary angiography in predicting acute coronary events.

## Introduction

Coronary artery disease (CAD) remains the foremost important cause of death worldwide. Preventing early deaths and the late consequences of myocardial infarction (MI) by a cost effective non-ionizing technique seems to be a reasonable part for the solution of this complex issue to our modern society [1].

In order to non-invasively detect CAD in patients with normal left ventricular function (LVF), avoiding unnecessary coronary angiography, stress echocardiography has been utilized successfully for many years. However, the spatial extent and severity of induced ischemia are important factors with regard to prognosis and, unfortunately, wall motion (WM) analysis by stress echocardiography does not accurately represent these factors [2–4].

Real-time myocardial perfusion echocardiography (RTMPE) has been demonstrated a useful technique for quantification of myocardial perfusion (MP) and determination of myocardial blood flow reserve (MBFR) [5–10]. Prognostic value of qualitative analysis of MP by RTMPE has been assessed using both dobutamine and high-dose dipyridamole stress echocardiography [11–13]. Although the accuracy of MBFR with a cut-off value of 2.0 has demonstrated to be accurate in the literature for detecting CAD [7, 14], the predictive importance of MBFR obtained by RTMPE was demonstrated only using vasodilator stress [15]. Therefore, the predictive importance of MBFR obtained by RTMPE using dobutamine stress is still not clear. Also, there is no data comparing the prognostic value of WM to qualitative and quantitative assessments of MP in patients with coronary angiography.

We sought to determine whether MBFR using dobutamine stress may add incremental prognostic value over conventional analysis of WM and MP in patients with known or suspected CAD and normal LVF.

## Methods

We prospectively studied 176 patients with known or suspected CAD who were referred for coronary angiography and underwent dobutamine-stress RTMPE. Inclusion criteria were: age ≥18 years, normal LVF (ejection fraction ≥50%). Exclusion criteria were previous coronary bypass graft surgery, unstable angina (UA), severe valvular disease, uncontrolled hypertension, ventricular arrhythmias, allergy to contrast, intracardiac shunt, pregnancy and breast-feeding. Median interval between RTMPE and coronary angiography was 17 days (1–52 days). The study protocol was approved by the ethical committee of Heart Institute—University of São Paulo Medical School and all patients gave written informed consent to participate. Quantitative coronary angiography was performed using a computerized system II CAAS (Cardiovascular Angiography Analysis System II, Netherlands). A significant stenosis was defined as one which reduced luminal diameter by ≥50%. Patients were divided into those with normal coronary arteries, those with a significant lesion in 1 coronary artery territory (CAT) and those with lesions in ≥2 CAT.

### RTMPE

A complete resting two-dimensional echocardiogram was performed, with special attention for the assessment of global and segmental left ventricular systolic function. Baseline left ventricular ejection fraction was calculated using modified Simpson’s rule [16] and the left ventricle was divided into 17 segments for both WM and MP analysis [17]. Commercially available ultrasound systems (Sonos 7500 and iE33- Philips Medical Systems, Bothell, Washington, USA) equipped with a S3 broadband transducer and contrast echo software with low-mechanical index imaging was used for RTMPE. Imaging was performed in the apical 2-, 3- and 4-chamber views using power modulation mode with a mechanical index of 0.2, frame rate 25–30 Hz, and continuous intravenous infusion of either lipid-encapsulated microbubbles Definity (Lantheus Medical Imaging, Inc., N. Billerica, Massachusetts, USA) or PESDA (perfluorocarbon-exposed sonicated dextrose albumin). The formulation of PESDA has been described elsewhere [18]. Microbubble destruction was achieved using a packet of five high-intensity (mechanical index 1.5) pulses (flash). Ultrasound contrast agent Definity was diluted in 60 mL saline solution and infused continuously into the right antecubital vein after an initial bolus dose of 3 mL. After that, infusion rates of 4.8 mL/min was started adjusting it according to the shadowing effect observed in the mid left atrial cavity in the four chamber view projection, never exceeding a dose of 10 mL/min. PESDA was prepared by diluting a suspension of 0.1 mL/Kg into a 80 mL of 5% dextrose, and administered intravenously in a continuous fashion at a rate of 2–5 mL/min [18]. Contrast infusion rate was adjusted till complete left ventricular cavity opacification and shadowing in the mid left atrial cavity was obtained. Once a stable myocardial enhancement was reached, the contrast infusion rate was kept constant, and sequences of low-power perfusion images containing at least 15 cardiac cycles after the flash were acquired. After completion of resting perfusion sequences, contrast infusion was stopped, and dobutamine infusion at a starting dose of 5μg. Kg^-1.^ min^-1^. This was followed by increasing doses of 10, 20, 30 to a maximal dose of 40μg. Kg^-1.^ min^-1^ in 3 minute stages. Atropine (up to 2 mg) was injected during the 20μg. Kg^-1.^ min^-1^ infusion, if heart rate was <100 beats/min [19]. Peak stress images were acquired in each of the three apical windows using the same flash impulse technique.

End points of dobutamine stress were achievement of target heart rate (85% of age predicted maximal heart rate), maximal dobutamine/atropine doses, development of severe or extensive WM abnormalities or intolerable side effects. Blood pressure and cardiac rhythm were continuously monitored prior, during and 20 minutes after dobutamine infusion. Twelve-lead electrocardiograms were obtained at baseline and at each 3-minute intervals during dobutamine infusion, and at any time at the operating physician’s discretion. The dobutamine stress was considered diagnostic if the target heart rate and/or inducible abnormalities were detected by qualitative analysis of WM or MP. The tests were considered nondiagnostic if the patients failed to achieve the target heart rate without inducible WM or MP abnormalities.

### Qualitative analysis of WM and MP

Qualitative analysis of segmental myocardial contraction was based on visual assessment of myocardial thickening during optimal cavity contrast enhancement and was graded according to a scoring system as follows: 1-normal, 2-hypokinesis, 3-akinesis and 4-dyskinesis. In order to maintain linearity with the MP analysis, the 17^th^ segment was also scored for WM when an abnormality was observed at the very edge of the left ventricular apex. A test was considered positive for WM analysis if new segmental WM abnormalities were present in one or more segments [20]. In the setting of resting segmental dysfunction, a segment was considered ischemic if it recovered during low dose dobutamine infusion, and then worsened by at least one score grade during peak dobutamine stress. Qualitative analysis of MP was based on a visual assessment of subendocardial and transmural myocardial contrast enhancement after the flash impulse. The myocardial opacification during this phase was graded visually as follows: grade 1, intense; grade 2, reduced; grade 3, lack of opacification in the first 4 to 5 cardiac cycles following the flash impulse. The reduced myocardial opacification (grade 2) could represent either reduced intensity at all times, and/or delayed appearance of contrast either compared to other segments or requiring >2 s during stress. It was defined as positive if one or more segments exhibited a new grade 2 or 3 defect in one or more myocardial segments at peak stress. Both WM and MP analysis were performed separately, by more than one month apart, by one experienced observer (WMJ) who was blinded to angiographic and prognostic findings.

### Quantitative analysis of RTMPE

Off-line image analysis for quantification was performed with a commercially available software (Q-lab 6.0 Phillips Medical Systems, Bothell, WA, USA). Regions of interest were traced manually within the 17 segments of myocardium and in the adjacent left ventricular cavity. Myocardial plateau (AM, dB) and adjacent left ventricular plateau (ALV, dB) signal intensities, and signal intensity exchange rate (ß/s)–that is proportional to myocardial blood flow velocity—were calculated automatically by the software. Normalized myocardial acoustic intensity (An) was defined as the ratio of AM divided by ALV, representing the relative myocardial blood volume (A, mL/mL) [8, 14, 21, 22]. To originate an index of blood flow, the product of An and ß (dB/dB/s) was calculated. For each study subject, MBFR (An x ß), and myocardial blood flow velocity reserve (ß) were calculated in each coronary artery territory as the arithmetic mean of values obtained individually in the segments assigned to each coronary artery territory.

The cutt-off values of ß reserve and MBFR that represent the points of best performance for accurately diagnose angiographically coronary obstruction have been previously reported in our laboratory and were used in this study to differentiate normal and abnormal ß reserve and MBFR [7]. The cutt-off value of ß reserve and MBFR were 2.0 and 2.6, respectively. These values were used for comparative analysis according to the classification of patients into those with normal coronary arteries, those with a significant lesion in one arterial territory and those with lesions in two or more arterial territories. Intraobserver variability of ß reserve and MBFR was assessed in 10 randomly assigned patients, by the same observer, with the two independent measurements made at least one year apart.

### Quantitative coronary angiography

Coronary angiography was performed by Judkins technique by femoral puncture using an equipment Integris H-3000 (Phillips, Holland) with digital processing of images. After the acquisition, the images were stored in an optical disc format or DICOM (Digital Imaging and Communication). Quantitative coronary angiography was performed using a computerized system II CAAS (Cardiovascular Angiography Analysis System II, Pie Medical Inc. Maastricht, Netherlands). A significant stenosis was defined as one which reduced luminal diameter by ≥ 50%. Patients were divided into those with normal coronary arteries, those with a significant lesion in one arterial territory and those with lesions in two or more arterial territories.

### Follow-up

Follow-up was obtained by review of the patient´s hospital chart, electronic records and telephone interview with the patients. Acute coronary events were defined as cardiac death, nonfatal myocardial infarction, and refractory or unstable angina requiring coronary revascularization during the same hospitalization. Cardiac death was defined as death associated with known or suspected myocardial infarction, life-threatening arrhythmia, or pulmonary edema. Sudden unexpected death occurring without another explanation was included as cardiac death. Nonfatal myocardial infarction was defined by means of a serial increase in cardiac specific enzymes and/or development of new ECG changes. Patients who underwent elective coronary revascularizations during the follow-up period for other indications (compelling anatomy, patient considered high risk for reasons other than refractory symptoms) were censored at the time of procedure, since the stress echocardiography results could have influenced the decision to revascularization. The date of an event was used to establish the time of follow-up, or in the absence of an event, the date of the last interview or review of medical records were used to determine it.

### Statistical analysis

Data were expressed as mean value ± standard deviation for continuous variables, and absolute (n) or relative (%) frequencies for discrete variables. Data normality was tested by D’Agostino-Pearson Omnibus test. Discrete variables were analyzed by Chi-square test or Fischer exact test, if necessary. Continuous variables among groups were analyzed by Student’s t-test or ANOVA (two or three groups, respectively, for variables with normal distribution) and Mann-Whitney or Kruskal-Wallis (two or three groups, respectively, for variables with distribution not considered normal). The Kaplan-Meier curves were used to estimate the distribution of time to the events. The Cox model was used to estimate the hazard ratio (HR) of events for each variable. The differences between the event curves were compared with the log-rank test. Clinical variables inserted in the model were age, male sex, ≥ risk factors for CAD (including hypertension, diabetes, cigarette smoking and familial history for CAD), stable angina, previous myocardial infarction and previous percutaneous coronary intervention. Parameters derived from dobutamine stress RTMPE included WM (normal or abnormal), qualitative MP (normal or abnormal), β reserve (normal when ≥ 2.0 or abnormal when < 2.0) and MBFR (normal when ≥ 2.6 or abnormal when < 2.6). Multivariate predictors of events were determined by the Cox proportional-hazards model. The incremental value of RTMPE information was assessed in 3 modeling steps. The first step consisted of fitting a multivariate model of qualitative analysis of WM only (normal versus abnormal). Qualitative analysis of MP by RTMPE was then added to this model (normal versus abnormal), followed by quantitative analysis of MP using β reserve (< 2.0 or ≥ 2.0) or MBFR (< 2.6 or ≥ 2.6). The significance of adding additional variables to previous modeling steps was based on the change in model-based likelihood statistics, with degrees of freedom equal to the number of additional variables. Intraobserver variability was assessed using the coefficient of variation and intraclass correlation coefficient. A two-tailed p value < 0.05 was considered statistically significant. Statistical tests were performed using software SPSS 13.0 for Windows (SPSS Inc., Chicago, Illinois, USA).

## Results

Among the 176 patients initially considered for enrollment, 8 patients were excluded because they did not complete dobutamine-stress RTMPE. The final study population was constituted by 168 patients. There were 91 men (54%), and the mean age was 58±8.7 years. Risk factors for CAD were diabetes mellitus in 63 (37.5%), systemic hypertension in 148 (88%), hypercholesterolemia in 140 (83.3%), and cigarette smoking in 35 (20.8%) patients. One hundred twenty-seven (76%) patients were taking beta-blockers, 119 (70.8%) statins, 144 (85.7%) aspirin and 43 (25.6%) calcium channel blockers. Twenty-eight (17%) patients had a history of a previous myocardial infarction and 17 (10%) had previous percutaneous interventions.

The contrast agent Definity was used in 94 (56%) patients and PESDA in 74 (44%). The mean maximal dose of dobutamine during the stress test was 30±6 μg/Kg/min. Atropine was infused in 158 (94%) patients. The mean percentage of maximal predicted heart rate achieved at peak stress was heart rate increased from 64±11 beats/min at baseline to 81±16 beats/min at peak stress (p<0.001). There were no significant changes from baseline to peak stress in systolic blood pressure (139±22 mm versus 132±23 mm Hg; p = NS) and diastolic blood pressure (78±12 mm Hg versus 75±14 mm Hg; p = NS). The stress test was accomplished without serious complications in all patients. No myocardial infarction or death occurred either during or immediately after dobutamine stress.

Follow-up data were available in all patients. Patients were followed for a median of 34 months (range, 5 days to 6.9 years). A total of 17 (10.1%) patients had acute coronary events during the follow-up (3 cardiac deaths, 3 nonfatal myocardial infarctions and 11 unstable angina). Acute coronary events occurred at a median of 9 months (range, 5 days to 47 months) after the dobutamine stress RTMPE. A total of 21 patients underwent elective coronary revascularizations during follow-up period for other reasons than unstable angina and the follow-up was censured at the time of procedure. Patients without events were followed-up for a minimum of 3 months.

### Qualitative analysis of RTMPE

**Fig 1:**
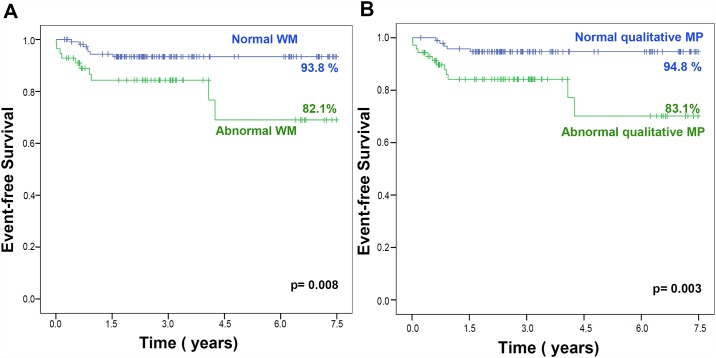
Kaplan-Meier curves of patients according the results of Wall Motion (WM) (A) and qualitative Myocardial Perfusion (MP) (B).

Resting WM was abnormal in at least one coronary artery territory in 28 patients (17%). All patients had normal resting ejection fraction. Inducible WM abnormalities were observed in 56 (33%) patients while MP abnormalities by qualitative analysis were observed in 85 (51%) patients. Patients with abnormal WM during dobutamine stress RTMPE had a higher proportion of acute coronary events (p = 0.019) during follow-up. Patients with abnormal MP by qualitative analysis also had a higher proportion of acute coronary events (p = 0.013) during follow-up. The Kaplan-Meier survival curves according to the results of WM and qualitative analysis of MP are illustrated in Fig 1. Note that event rates were significantly different between patients with normal and abnormal WM (p = 0.008) and normal and abnormal qualitative MP (p = 0.003). At the end of follow-up, event-free survival rate was 94.8% for patients with normal qualitative MP, 93.8% for patients with normal WM, 83.1% for patients with abnormal qualitative MP and 82.1% for patients with abnormal WM.

### Quantitative analysis of RTMPE

**Fig 2:**
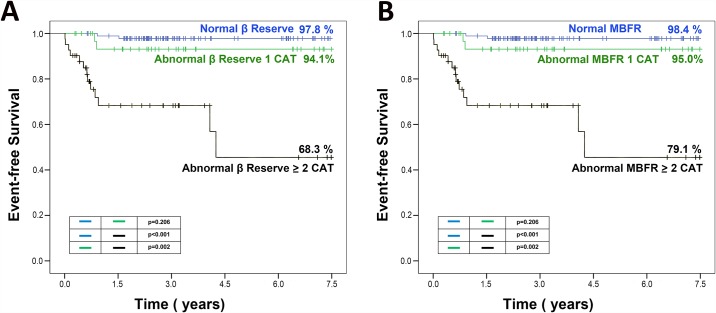
Kaplan-Meier curves of patients according the results of β reserve (A) and MBFR (B).

Quantitative analysis of RTMPE was performed by coronary artery territories and by patients. The coefficient of variation for β reserve was 10.7%, with intraclass correlation coefficient of 0.94 (95% confidence interval = 0.88–0.97). The coefficient of variation for MBFR reserve was 11.6%, with intraclass correlation coefficient of 0.97 (95% confidence interval = 0.94–0.99). Among the 168 patients, 28 (16.7%) had abnormal β reserve in only one coronary artery territory and 66 (39%) in ≥2 territories. The characteristics of patients according to β results are presented in Table 1. The Kaplan-Meier survival curves according to the results of β reserve are illustrated in Fig 2A. A total of 40 (24%) patients had abnormal MBFR in 1 territory and 79 (47%) with abnormal MBFR in ≥2 territories. The characteristics of patients according to MBFR results are presented in Table 1. Kaplan-Meier survival curves according to the results of MBFR are illustrated in Fig 2B.

**Table 1:** Characteristics of patients according to the results of βreserve and MBFR.

**Variables**	**Normal β**	**Abnormal β 1 CAT**	**Abnormal β ≥ 2 CAT**
Age (years)	57.5±8.3	59.6±9.0	59.9±9.1
Male	43(46.2%)	19(55.9%)	29(70.7%)†
≥3 risk factors	57(61.3%)	27(79.4%)	23(56.1%)
Stable angina	40(43.0%)	21(61.8%)	24(58.5%)
Previous MI	6(6.5%)	5(14.7%)	17(41.5%)†¶
Diabete Mellitus	33(35.5%)	10(29.4%)	20(48.8%)
LVDD	46±5	48±5	50±5
LVWT	10±2	10±2	10±1
Abnormal WM	8(8.6%)	18(52.9%)*	30(73.2%)†
Abnormal QMP	13(14.0%)	23(67.6%)*	35(85.4%)†
Events	2(2.2%)	2(5.9%)	13(31.7%)†¶
**Variables**	**Normal MBFR**	**Abnormal MBFR 1CAT**	**Abnormal MBFR ≥ 2CAT**
Age (years)	58.8±7.8	56.2±8.9	59.7±9.1
Male	25(41.0%)	23(57.5%)	43(64.2%)†
≥3 risk factors	37(60.7%)	26(65.0%)	44(65.7%)
Stable angina	27(44.3%)	22(55.0%)	36(53.7%)
Previous MI	3(4.9%)	5(12.5%)	20(29.9%)†
Diabetes Mellitus	21(34.4%)	13(32.5%)	29(43.3%)
LVDD	46±5	49±5	49±5
LVWT	10±2	10±2	10±1
Abnormal WM	3(4.9%)	12(30.0%)*	41(61.2%)†¶
Abnormal QMP	7(11.5%)	14(35.0%)*	50(74.6%)†¶
Events	1(1.6%)	2(5.0%)	14(20.9%)†

CAT = coronary artery territory; MBFR = myocardial blood flow reserve; MI = myocardial infarction; PCI = percutaneous coronary intervention; QMP = qualitative myocardial perfusion; LVDD = left ventricular diastolic diameter; LVWT = left ventricular wall thickness; WM = wall motion.

*p<0.05 between normal reserve and abnormal reserve in 1CAT;

^†^p<0.05 between normal reserve and abnormal reserve in ≥2 CAT;

^¶^p<0.05 between abnormal reserve in 1CAT and ≥2 CAT

**Fig 3:**
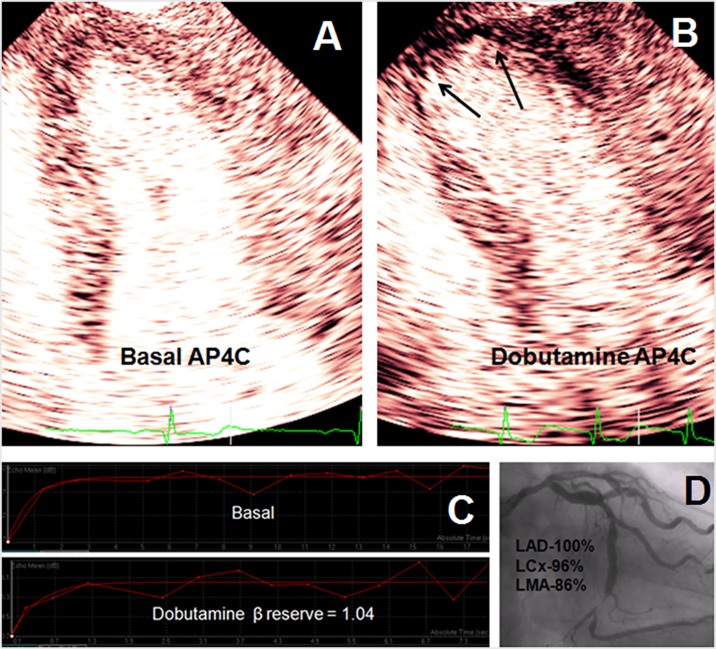
Apical four-chamber view imagin of a 67 year-old man with normal wall motion and qualitative myocardial perfusion at rest (**A)**. **(B)** During dobutamine-stress, it was observed apical dyskinesis and marked perfusion defect (arrow). **(C)** Acoustic intensity curves at rest and during stress demonstrated a low β reserve. **(D)** Coronary angiography demonstrated significant coronary artery disease. Patient had event after 8 months of echocardiography. LAD-left anterior descending artery, LCx- left circumflex artery, LMA- left marginal artery.

Fig 3 illustrates examples of RTMPE imaging at rest and during dobutamine stress with respective curves of myocardial blood flow quantification in patients with and without events during the follow-up.

### Coronary angiography

Among the 168 studied patients, 31 (18%) had coronary stenosis in only 1 territory and 55 (33%) had abnormal coronary artery in ≥2 territories. Kaplan-Meier survival curves according to the results of MBFR are illustrated in Fig 2B.

### Predictors of events

Table 2 presents the univariate and multivariate predictors of events. By univariate analysis, WM, qualitative MP and quantitative parameters obtained by RTMPE were predictors of events. However, by multivariate analysis, the only independent predictors of events were abnormal β reserve and abnormal MBFR (HR = 21, 95% CI = 4.5–99; p<0.001 and HR = 15.8, 95% CI = 2–124; p = 0.009, respectively) when they were abnormal in ≥2 territories. After adding coronary angiography data to the multivariate analysis model, β reserve was still the only predictor of acute coronary events with OR = 21 (95% CI = 4.5–99), p<0.001.

**Table 2:** Predictors of acute coronary events by univariate and multivariate analysis.

Variables	Univariate analysis		Multivariate analysis	
	HR (95%CI)	p	HR (95%CI)	p
Age (years)	-	NS	-	NS
Male sex	-	NS	-	NS
≥3 risk factors	-	NS	-	NS
Stable angina	-	NS	-	NS
Previous myocardial infarction	-	NS	-	NS
Previous percutaneous coronary intervention	-	NS	-	NS
Left ventricular diastolic diameter	-	NS	-	NS
Left ventricular wall thickness	-	NS	-	NS
Abnormal wall motion	3.2(1.1–9)	0.024	-	NS
Abnormal qualitative myocardial perfusion	3.7(1.2–11)	0.018	-	NS
Abnormal MBFR 1CAT	3.1(0.27–36)	0.355	-	NS
Abnormal MBFR ≥2CAT	15.8(2–124)	0.009	15.8(2–124)	0.009
Abnormal β reserve 1CAT	2.8(0.3–21)	0.306	-	NS
Abnormal β reserve ≥2CAT	21(4.5–99)	<0.001	21(4.5–99)	<0.001

CAT = coronary artery territory; MBFR = myocardial blood flow reserve

**Fig 4:**
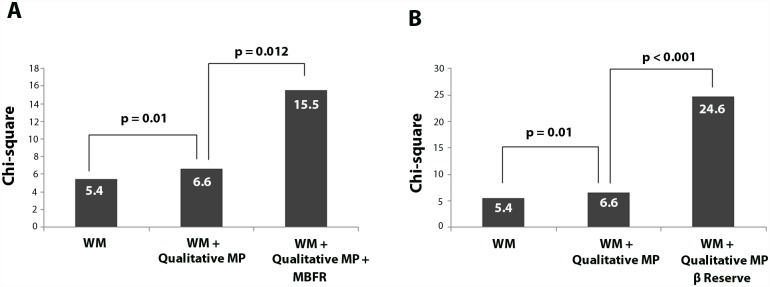
Incremental value of abnormal Myocardial Perfusion (MP), abnormal Myocardial Blood Flow Reserve (MBRF) (A) and abnormal β reserve (B) over abnormal Wall Motion (WM) using a Cox model for predicting acute coronary events.

Sequential Cox regression models were fit to test the incremental value of quantitative parameters of RTMPE over WM and qualitative analysis of MP (Fig 4A). The presence abnormal MP, by qualitative analysis, increased the likelihood of acute coronary events over the analysis of WM (χ2 = 5.4 and χ2 = 6.6, respectively; p = 0.010). Abnormal MBFR added significant incremental value in predicting acute coronary events over both the qualitative analysis of WM and MP (χ2 = 6.6 and χ2 = 15.5, respectively; p = 0.012). However, abnormal β reserve added further significant incremental value in predicting acute coronary events over both the qualitative analysis of WM and MP (χ2 = 6.6 and χ2 = 24.6, respectively; p 0.001). Incremental value (expressed as Chi-square values with incremental degrees of freedom) of abnormal β reserve over abnormal WM, qualitative MP and coronary angiography are displayed in Fig 4B.

**Fig 5:**
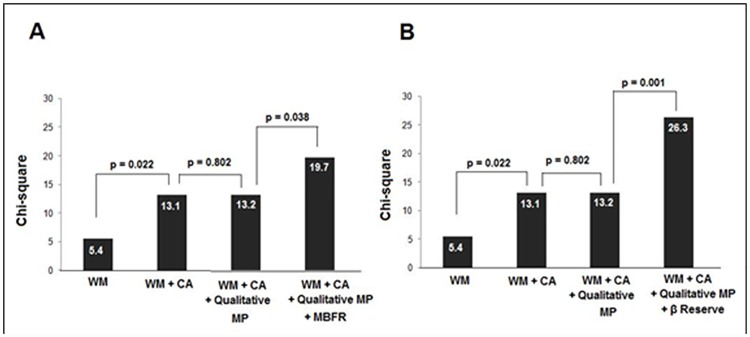
Incremental value of Coronary Angiography (CA), abnormal Myocardial Perfusion (MP), abnormal Myocardial Blood Flow Reserve (MBFR) (A) and abnormal β reserve (B) over abnormal Wall Motion (WM) using a Cox model.

The presence abnormal β reserve in ≥2 territories increased the likelihood of acute coronary events among all variables (χ2 from 13.2 to 26.3; p = 0.001, Fig 5B). Using this model, abnormal MBFR in ≥ 2 territories also increased the likelihood of acute coronary events over the analysis of WM, coronary angiography and qualitative analysis of MP (χ2 from 13.2 to 19.7; p = 0.038, Fig 5A).

## Discussion

Myocardial contrast echocardiography has been shown to be useful for the non-invasive assessment of myocardial perfusion [23–28]. Additionally, the diagnostic accuracy for the detection of CAD by quantitative RTMPE has been examined, and has consistently demonstrated enhanced diagnostic performance, especially when compared to qualitative analysis of WM and MP [7, 14]. Although time consuming, the advantage of quantitative RTMPE is that it uses no ionizing agent, and thus permits a safe, cost-effective analysis of myocardial blood flow changes during stress testing. This study provides important new information regarding the prognostic value of quantitative myocardial contrast echocardiography during stress testing. We evaluated only patients with known or suspected CAD and preserved left ventricular systolic function. There is already a large myriad of excellent prognostic information in patients with left ventricular dysfunction, while accurate risk assessments of patients with normal resting left ventricular systolic function is more challenging. This study was the first to demonstrate that quantitative RTPME may provide an independent predictor of events and has incremental additional prognostic value over clinical variables, WM, qualitative MP and coronary angiography in patients with normal resting systolic function.

The accurate risk stratification of patients with CAD has become of ultmost importance for guiding clinical management. Nuclear scintigraphy is routinely used for prognostic evaluation of patients with known or suspected CAD, with several studies envolving high number of patients demonstrating its value for noninvasive prediction of events [29–30]. However, concerns regarding the use of radiation are still a problem. Previous studies have been shown, in a heterogenous population of patients with known or suspected CAD, that qualitative analysis of MP during dobutamine stress RTMPE provided independent information for predicting hard events [11, 31–33]. Tsutsui et al. evaluated the prognostic value of qualitative analysis of RTMPE in 788 patients during dobutamine stress echocardiography using commercially available contrast agents and demonstrated that abnormal MP had significant incremental value over clinical factors, resting ejection fraction, and WM responses in predicting death and nonfatal myocardial infarction (p = 0.001) [11]. Miszalski-Jamka et al have found similar results with RTMPE during supine bicycle stress echocardiography. While left ventricular opacification did not improve the prognostic value of 2-dimensional stress echocardiography, MP had incremental prognostic value over WM analysis for the prediction of cardiac death, nonfatal myocardial infarction and revascularization during long term follow-up [32].

Basic et al compared the prognostic value of myocardial contrast echocardiography using dipyridamole and technetium-99m sestamibi single photon emission computed tomography (SPECT)-myocardial perfusion imaging in 51 patients with known or suggested CAD [33]. The authors demonstrated that both techniques presented equivalent event-free survival. A normal myocardial contrast echocardiography perfusion study was able to accurately identify those patients at low risk for a cardiac event, and provided identical information when compared with SPECT. On the other hand, patients with abnormal myocardial contrast echocardiography perfusion study, the cardiac event rate in the follow-up period was 29%, which was similar to the 25% cardiac event rate found using SPECT. In another study comparing myocardial contrast echocardiography with SPECT [31] the authors have found that dipyridamole-stress myocardial contrast echocardiography provided powerful prognostic information that is superior to clinical variables, electrocardiography, left ventricular systolic function, WM analysis, and SPECT in a high number of patients (n = 261) with known or suspected CAD. Of note, all these studies performed a qualitative analysis of myocardial perfusion.

In the present study, we studied a selected population of patients with preserved ventricular function, and also found that qualitative analysis of MP was predictive of acute coronary events. However, quantitative analysis of MP was even more predictive, having independent predictive value over all clinical, echocardiographic, and angiographic variables. Previously, our group found a cut-off value for β reserve of 2.0 was optimal for detecting angiographically significant CAD [7], and here we demonstrated that this value is also an excellent predictor of prognosis.

In multivariate analysis, abnormal β reserve in two or more coronary territories was the best independent predictor of events. This finding defines that a larger spatial distribution of reduced MBFR identifies patients at higher risk. This is consistent with qualitative data already established in the literature for other stress echocardiographic modalities [4, 11, 34, 35] and nuclear scintigraphy. However, our study shows the superiority of the β parameter, in predicting outcome. Coronary angiography is known to be the reference standard for detection of coronary stenoses, but its ability to predict those at risk for acute coronary syndromes is poor [36]. Furthermore, it is an invasive procedure that uses a significant amount of ionizing radiation and a nephrotoxic contrast agent. RTMPE has the advantage of being noninvasive test that does not require ionizing radiation. The current study demonstrated the potential for quantitative RTMPE to best identify those at risk for acute coronary syndromes. Further study is needed to examine what role specific therapeutic interventions (revascularization, intensive medical therapy) have on the predictive value of quantitative RTMPE.

### Limitations of the study

Since patients in this study were already undergoing coronary angiography, we may have selected those at the greatest clinical risk. However, nearly half of the patients still did not have an angiographically significant coronary stenosis. Furthermore, patients who had “compelling anatomy” and had percutaneous revascularization at the time of the diagnostic angiogram were also excluded. This resulted in an actual event rate of only 10% over the approximate three year follow-up period. Therefore, the population studied was similar to what would be expected for patients presenting for stress echocardiography. This study was a single center study, with a vast experience in performing RTMPE. Although this may have affected the results of the qualitative assessments of MP, it probably had little impact on the quantitative assessments of MBFR. The quantitative assessments of perfusion are time consuming, and require proper placement of the regions of interest, but less prone to subjectivity or operator experience. Nonetheless, multicenter trials will be required to determine the ability of quantitative assessments of MBFR to predict outcome during dobutamine stress echocardiography in a broader setting. A concerted effort of the different ultrasound manufacturers is also needed, to standardize the quantitative measurements, and assist in developing normal databases as well as a more eficiente, used-friendly quantification software.

## Conclusion

Quantification of myocardial blood flow velocity reserve during dobutamine stress RTMPE is an independent predictor of acute coronary events in patients with known or suspected CAD with preserved left ventricular function, and provides additional prognostic information over clinical variables, qualitative analysis of WM and MP, and coronary angiography data. Quantitative parameters identify a subgroup of individuals at a higher risk of cardiac events, especially when the abnormal flow reserve involves more than one coronary artery territories.

## Supporting information

S1 Table Clinical data and real-time myocardial perfusion quantification. (PDF)
