# Supplementary material for: Prognostic value of dobutamine stress myocardial perfusion echocardiography in patients with known or suspected coronary artery disease and normal left ventricular function
Source: PLoS One. 2017 Feb 24;12(2):e0172280. doi: 10.1371/journal.pone.0172280 (PMC5325237; doi:10.1371/journal.pone.0172280)
Supplement: S1 Table — (PDF) [file pone.0172280.s001.pdf]

| patient | Idade | Sexo | ≥ 3FR | DM | Angina Est | IAM P | ATC P | Nitrato | Bloq Ca | AAS | Beta-bloq |
|---|---|---|---|---|---|---|---|---|---|---|---|
| 1 | 40 | M | N | S | S | N | N | N | N | N | N |
| 2 | 46 | F | S | N | S | S | N | N | S | S | S |
| 3 | 42 | F | S | N | N | N | N | S | N | S | S |
| 4 | 61 | F | S | N | N | S | N | S | N | S | S |
| 5 | 64 | F | S | S | S | N | N | S | N | S | N |
| 6 | 78 | F | N | S | S | N | N | N | N | S | S |
| 7 | 64 | F | S | N | N | N | N | N | N | S | S |
| 8 | 49 | F | S | S | S | N | N | N | N | S | S |
| 9 | 53 | F | S | S | S | N | N | S | N | S | S |
| 10 | 44 | M | N | N | N | N | N | N | N | S | S |
| 11 | 54 | F | N | N | N | N | N | N | N | N | N |
| 12 | 52 | M | S | N | S | N | N | S | S | S | S |
| 13 | 35 | M | N | N | S | N | N | N | N | S | S |
| 14 | 49 | M | S | N | N | N | N | N | N | N | S |
| 15 | 59 | M | N | N | S | N | S | S | N | S | S |
| 16 | 56 | M | N | N | N | N | N | N | N | N | N |
| 17 | 72 | M | S | N | N | N | S | S | S | S | S |
| 18 | 52 | M | N | N | S | N | N | N | N | N | N |
| 19 | 59 | M | S | S | N | N | S | N | S | S | S |
| 20 | 50 | M | S | N | N | N | N | S | S | S | N |
| 21 | 60 | M | S | N | N | N | N | N | N | S | N |
| 22 | 49 | M | N | N | N | S | N | N | N | S | S |
| 23 | 65 | M | S | N | S | S | N | S | N | S | S |
| 24 | 59 | M | S | S | S | N | N | N | N | S | S |
| 25 | 55 | M | S | S | N | N | N | N | N | S | S |
| 26 | 57 | M | S | S | N | S | S | N | S | S | S |
| 27 | 67 | F | S | S | S | N | N | N | S | S | S |
| 28 | 71 | M | S | N | S | N | N | N | N | S | S |
| 29 | 58 | F | S | S | S | S | N | N | N | N | N |
| 30 | 68 | M | S | S | S | N | N | S | N | S | S |
| 31 | 51 | M | S | S | S | N | N | N | S | S | N |
| 32 | 67 | M | N | N | S | N | N | S | S | S | S |
| 33 | 53 | M | S | N | N | N | N | N | S | S | S |
| 34 | 55 | F | S | S | S | N | N | N | S | S | S |
| 35 | 70 | F | S | N | N | N | N | S | N | S | N |
| 36 | 48 | M | S | N | N | N | N | N | N | S | S |
| 37 | 68 | F | S | S | N | N | N | S | S | S | S |
| 38 | 70 | F | N | N | S | N | N | N | N | S | S |
| 39 | 68 | M | S | S | N | S | N | N | S | S | N |
| 40 | 67 | F | S | S | S | N | S | N | S | S | S |
| 41 | 49 | M | N | N | S | N | N | N | S | S | N |
| 42 | 63 | M | N | N | N | N | N | N | N | S | S |
| 43 | 67 | M | S | N | S | S | N | N | S | S | S |
| 44 | 65 | M | S | N | S | N | N | N | N | S | N |
| 45 | 53 | M | N | N | N | N | S | S | N | N | S |
| 46 | 53 | F | S | S | N | N | N | N | S | S | N |
| 47 | 76 | F | S | S | S | N | N | S | N | S | S |
| 48 | 61 | F | S | N | N | N | N | N | N | N | S |
| 49 | 66 | F | S | S | S | N | N | N | N | S | S |

| | | | | | | | | | | | |
|---|---|---|---|---|---|---|---|---|---|---|---|
| **50** | 53 | F | S | N | S | N | N | N | N | S | N |
| **51** | 64 | M | N | S | S | N | S | N | N | S | S |
| **52** | 51 | M | S | S | S | N | N | N | N | S | S |
| **53** | 55 | M | S | S | N | N | N | S | N | S | S |
| **54** | 59 | M | S | N | N | S | S | S | N | S | S |
| **55** | 63 | M | S | N | S | S | N | N | N | S | S |
| **56** | 75 | M | N | N | S | N | N | S | N | S | S |
| **57** | 62 | M | S | S | S | S | N | S | S | S | N |
| **58** | 59 | F | S | N | S | N | N | S | N | S | S |
| **59** | 52 | M | N | S | S | S | N | N | N | S | S |
| **60** | 71 | F | S | N | N | N | N | S | N | S | N |
| **61** | 63 | F | S | S | S | N | N | N | S | S | S |
| **62** | 54 | F | S | N | N | N | S | N | N | N | N |
| **63** | 54 | M | S | S | N | N | N | N | N | S | S |
| **64** | 53 | F | N | N | S | N | N | N | N | S | N |
| **65** | 57 | M | N | N | N | N | N | N | N | S | S |
| **66** | 58 | F | S | N | N | N | N | N | S | S | S |
| **67** | 67 | F | S | N | N | N | S | S | N | S | N |
| **68** | 53 | F | S | N | S | N | N | N | S | S | N |
| **69** | 58 | F | S | N | N | N | N | N | N | N | S |
| **70** | 51 | M | S | N | N | N | N | N | N | S | S |
| **71** | 67 | F | S | N | S | N | N | S | N | S | N |
| **72** | 72 | F | S | S | S | N | S | S | N | S | S |
| **73** | 62 | M | N | N | N | S | S | S | N | S | S |
| **74** | 62 | F | N | N | S | N | N | N | N | S | S |
| **75** | 44 | M | S | S | N | S | N | N | N | N | S |
| **76** | 65 | M | S | S | S | N | N | N | N | S | N |
| **77** | 72 | M | N | N | S | N | N | N | N | S | N |
| **78** | 62 | M | N | N | S | N | N | N | N | S | S |
| **79** | 52 | M | S | N | N | S | N | N | N | S | S |
| **80** | 51 | M | S | N | N | N | N | N | S | S | S |
| **81** | 60 | M | S | N | S | N | N | N | N | S | S |
| **82** | 56 | M | S | S | S | N | N | S | N | S | S |
| **83** | 51 | M | N | N | N | N | N | N | N | S | S |
| **84** | 43 | M | S | N | N | N | N | N | N | S | S |
| **85** | 64 | M | S | S | N | N | N | S | S | S | S |
| **86** | 46 | M | N | S | N | N | N | S | N | S | S |
| **87** | 68 | M | S | N | N | N | N | N | N | S | S |
| **88** | 59 | M | S | S | N | S | N | N | N | S | N |
| **89** | 42 | M | N | N | N | N | N | N | N | S | S |
| **90** | 46 | M | N | N | S | N | N | N | N | N | S |
| **91** | 63 | M | S | N | N | N | N | N | N | N | S |
| **92** | 53 | F | S | N | N | N | N | N | S | S | S |
| **93** | 64 | F | S | N | S | N | N | S | N | S | S |
| **94** | 50 | M | S | S | S | N | N | N | N | S | N |
| **95** | 62 | M | S | S | N | N | N | N | N | S | S |
| **96** | 47 | M | S | N | N | N | N | N | N | N | N |
| **97** | 52 | F | S | N | S | N | N | N | N | S | S |
| **98** | 44 | M | S | S | N | N | N | N | N | S | N |
| **99** | 50 | F | N | S | S | N | S | S | N | S | S |

| | | | | | | | | | | | | |
|---|---|---|---|---|---|---|---|---|---|---|---|---|
| **100** | 64 | M | S | S | N | N | N | N | S | S | S |
| **101** | 78 | F | N | S | N | N | N | N | N | S | S |
| **102** | 68 | M | S | S | N | N | N | N | N | S | S |
| **103** | 64 | M | S | S | N | N | N | N | S | S | N |
| **104** | 57 | M | S | N | N | N | N | S | N | S | S |
| **105** | 49 | M | N | N | N | N | N | N | N | N | S |
| **106** | 69 | F | S | S | S | N | N | S | S | S | S |
| **107** | 69 | F | S | S | S | N | N | N | N | N | S |
| **108** | 56 | M | N | N | S | N | N | N | N | N | N |
| **109** | 61 | M | N | N | S | N | S | S | N | S | S |
| **110** | 60 | F | S | S | S | N | N | N | S | S | N |
| **111** | 46 | F | S | N | S | N | N | S | S | S | S |
| **112** | 62 | F | N | S | N | N | N | S | N | S | S |
| **113** | 51 | F | S | N | S | N | N | N | N | S | S |
| **114** | 57 | F | S | N | S | S | N | S | N | S | S |
| **115** | 53 | F | S | N | S | N | N | S | N | S | S |
| **116** | 75 | F | N | N | N | N | N | S | N | S | S |
| **117** | 58 | F | S | S | N | N | N | N | N | S | S |
| **118** | 71 | F | N | S | S | N | N | N | N | S | S |
| **119** | 56 | F | S | S | N | N | N | N | N | N | N |
| **120** | 46 | F | N | N | N | N | N | S | N | S | S |
| **121** | 63 | F | N | S | S | N | N | N | N | S | N |
| **122** | 63 | F | S | S | N | S | N | S | S | S | S |
| **123** | 43 | F | S | N | S | N | N | S | N | S | S |
| **124** | 57 | F | N | N | N | N | N | N | S | N | S |
| **125** | 56 | F | S | S | N | N | N | N | S | S | S |
| **126** | 62 | F | N | N | N | N | N | S | S | S | N |
| **127** | 57 | F | N | N | S | N | N | S | N | N | S |
| **128** | 60 | F | S | N | N | N | N | S | S | S | S |
| **129** | 75 | M | S | N | S | N | N | S | N | S | N |
| **130** | 63 | F | N | S | S | N | N | N | S | S | N |
| **131** | 58 | F | N | S | N | S | N | S | N | S | S |
| **132** | 64 | M | S | S | N | N | N | N | N | S | S |
| **133** | 56 | F | N | S | N | N | N | N | N | N | S |
| **134** | 69 | F | N | S | S | N | N | N | N | S | S |
| **135** | 72 | M | N | N | N | N | N | N | N | N | S |
| **136** | 52 | F | N | N | S | S | N | S | N | S | S |
| **137** | 65 | M | N | S | N | S | N | S | S | S | S |
| **138** | 51 | M | S | N | S | S | N | N | S | S | S |
| **139** | 70 | F | N | N | S | N | N | N | N | S | N |
| **140** | 60 | F | S | S | N | N | N | N | S | S | N |
| **141** | 67 | M | S | S | S | N | N | N | N | S | S |
| **142** | 58 | M | N | N | S | S | N | S | S | S | S |
| **143** | 54 | M | N | N | S | S | S | N | S | S | S |
| **144** | 57 | M | S | N | S | S | N | N | N | N | S |
| **145** | 57 | M | S | N | S | N | N | S | S | S | S |
| **146** | 42 | M | S | N | S | N | N | S | N | S | S |
| **147** | 40 | M | S | N | N | N | N | N | N | S | S |
| **148** | 72 | M | N | N | N | N | N | S | N | S | N |
| **149** | 59 | M | N | N | S | S | S | N | N | S | S |

| | | | | | | | | | | | | |
|---|---|---|---|---|---|---|---|---|---|---|---|---|
| **150** | 59 | F | N | N | S | N | N | S | N | S | S |
| **151** | 50 | M | S | N | N | N | N | N | N | S | S |
| **152** | 66 | M | N | N | S | N | N | S | N | S | S |
| **153** | 74 | F | N | N | S | N | N | S | S | S | S |
| **154** | 64 | M | N | S | S | N | N | N | N | S | S |
| **155** | 60 | M | S | S | N | N | N | N | N | S | S |
| **156** | 59 | F | N | N | N | N | N | S | N | S | S |
| **157** | 64 | M | N | N | N | N | S | S | N | S | S |
| **158** | 47 | F | S | N | N | N | N | S | N | S | S |
| **159** | 59 | F | S | N | S | N | N | N | N | S | S |
| **160** | 62 | M | S | N | S | S | N | S | S | S | S |
| **161** | 51 | F | S | N | N | N | N | N | N | S | S |
| **162** | 48 | F | S | N | S | N | N | S | N | S | S |
| **163** | 46 | F | S | N | N | N | N | N | N | S | S |
| **164** | 61 | F | N | N | S | N | N | N | N | N | N |
| **165** | 60 | F | N | N | N | N | N | N | N | S | S |
| **166** | 63 | M | S | N | N | N | N | N | N | S | S |
| **167** | 65 | M | S | S | S | S | N | N | N | S | S |
| **168** | 61 | F | N | N | N | N | N | N | N | S | N |

| Estatina | FE (S) | DDVE | DSVE | AE | SIV | PP | DIA | Fluxos | FC b | PAS b | PAD b | FC atingi( | % FC max | PAS p |
|---|---|---|---|---|---|---|---|---|---|---|---|---|---|---|
| N | 63 | 54 | 34 | 42 | 9 | 9 | Nor | Normal | 80 | 180 | 100 | 154 | 85 | 180 |
| S | 60 | 53 | 36 | 40 | 11 | 10 | Pse | Pseudon( | 53 | 131 | 76 | 121 | 70 | 186 |
| N | 68 | 40 | 24 | 26 | 8 | 8 | Nor | Normal | 80 | 140 | 100 | 169 | 95 | 170 |
| S | 60 | 53 | 34 | 38 | 9 | 8 | IM r | IM moder | 70 | 107 | 59 | 87 | 55 | 131 |
| S | 63 | 45 | 28 | 38 | 11 | 11 | Nor | Normal | 62 | 150 | 80 | 136 | 87 | 180 |
| S | 73 | 47 | 30 | 44 | 12 | 12 | IM ( | IM discre | 58 | 180 | 80 | 139 | 98 | 130 |
| S | 72 | 46 | 30 | 36 | 9 | 8 | AR\ | ARV | 46 | 120 | 70 | 138 | 88 | 140 |
| S | 69 | 42 | 26 | 39 | 9 | 8 | Nor | ARV | 59 | 134 | 76 | 132 | 77 | 145 |
| S | 68 | 46 | 30 | 38 | 10 | 10 | Nor | Normal | 56 | 131 | 72 | 150 | 89 | 136 |
| S | 69 | 49 | 33 | 31 | 9 | 9 | Nor | Normal | 58 | 120 | 70 | 150 | 85 | 179 |
| N | 64 | 40 | 26 | 32 | 7 | 7 | Normal | | 71 | 120 | 70 | 152 | 91 | 140 |
| S | 63 | 52 | 37 | 45 | 13 | 12 | ARV | | 60 | 150 | 80 | 69 | 41 | 210 |
| S | 70 | 47 | 30 | 37 | 10 | 8 | Nor | Normal | 62 | 110 | 80 | 163 | 88 | 150 |
| S | 65 | 49 | 30 | 42 | 10 | 10 | Normal | | 47 | 100 | 70 | 153 | 89 | 130 |
| S | 63 | 47 | 31 | 39 | 9 | 9 | ARV | | 41 | 120 | 80 | 123 | 73 | 140 |
| N | 65 | 55 | 36 | 42 | 10 | 9 | IM r | IM moder | 73 | 140 | 90 | 152 | 92 | 150 |
| S | 70 | 42 | 26 | 40 | 9 | 9 | IM ( | IM discre | 80 | 150 | 90 | 140 | 94 | 100 |
| S | 65 | 43 | 27 | 30 | 10 | 10 | AR\ | ARV | 75 | 130 | 82 | 160 | 95 | 140 |
| S | 68 | 50 | 31 | 41 | 11 | 10 | ARV | | 58 | 110 | 72 | 148 | 92 | 133 |
| S | 70 | 43 | 27 | 37 | 10 | 10 | ARV | | 70 | 120 | 90 | 149 | 87 | 150 |
| S | 49 | 45 | 34 | 34 | 9 | 9 | ARV | | 60 | 150 | 90 | 157 | 98 | 130 |
| S | 50 | 58 | 46 | 40 | 11 | 9 | Pseudonorma | | 63 | 113 | 74 | 154 | 90 | 169 |
| N | 50 | 56 | 38 | 45 | 12 | 8 | AR\ | ARV/Ac i | 63 | 120 | 70 | 142 | 92 | 140 |
| N | 67 | 48 | 30 | 42 | 12 | 12 | ARV | | 66 | 130 | 70 | 158 | 98 | 160 |
| S | 61 | 52 | 35 | 40 | 11 | 11 | AR\ | ARV | 58 | 120 | 80 | 153 | 93 | 130 |
| S | 60 | 59 | 34 | 46 | 12 | 11 | ARV/hipoc inf | | 70 | 148 | 91 | 141 | 87 | 181 |
| S | 66 | 48 | 30 | 42 | 11 | 12 | ARV | | 58 | 130 | 80 | 125 | 82 | 130 |
| S | 59 | 51 | 35 | 41 | 12 | 12 | ARV/ AC bas | | 44 | 131 | 71 | 120 | 80 | 156 |
| N | 48 | 54 | 33 | 28 | 9 | 9 | Acinesia apic | | 79 | 140 | 90 | 150 | 97 | 160 |
| N | 70 | 46 | 25 | 34 | 13 | 12 | Pseudonorma | | 58 | 147 | 65 | 145 | 95 | 164 |
| S | 68 | 44 | 27 | 34 | 9 | 9 | AR\ | ARV | 76 | 123 | 91 | 147 | 86 | 177 |
| S | 68 | 51 | 36 | 42 | 10 | 9 | AR\ | ARV | 50 | 121 | 70 | 133 | 87 | 164 |
| N | 69 | 44 | 27 | 41 | 14 | 13 | AR\ | ARV/IM c | 53 | 153 | 87 | 137 | 82 | 147 |
| S | 68 | 47 | 32 | 33 | 13 | 12 | ARV | | 83 | 160 | 100 | 165 | 100 | 180 |
| N | 68 | 47 | 28 | 34 | 12 | 12 | AR\ | ARV | 84 | 150 | 80 | 157 | 100 | 180 |
| S | 75 | 52 | 33 | 34 | 8 | 8 | nor | Normal | 63 | 140 | 85 | 146 | 85 | 197 |
| S | 64 | 51 | 33 | 44 | 12 | 11 | Pseudonorma | | 64 | 140 | 90 | 131 | 86 | 180 |
| S | 66 | 41 | 27 | 34 | 11 | 11 | AR\ | ARV | 52 | 150 | 70 | 132 | 88 | 180 |
| S | 60 | 58 | 33 | 50 | 12 | 12 | Pseudonorma | | 98 | 150 | 87 | 158 | 104 | 180 |
| S | 66 | 43 | 25 | 35 | 8 | 8 | ARV | | 62 | 140 | 60 | 152 | 99 | 159 |
| S | 60 | 49 | 29 | 42 | 12 | 12 | IM ( | IM discre | 74 | 110 | 80 | 158 | 92 | 90 |
| S | 68 | 49 | 32 | 41 | 9 | 9 | AR\ | ARV | 43 | 150 | 90 | 136 | 86 | 140 |
| N | 62 | 47 | 30 | 36 | 10 | 10 | ARV | | 48 | 120 | 70 | 114 | 74 | 110 |
| S | 70 | 51 | 30 | 40 | 11 | 10 | Pseudonorma | | 51 | 128 | 67 | 156 | 101 | 172 |
| N | 64 | 41 | 26 | 34 | 8 | 8 | Normal | | | | | | | |
| N | 70 | 44 | 28 | 38 | 10 | 9 | ARV | | 92 | 130 | 89 | 156 | 93 | 156 |
| S | 71 | 45 | 27 | 37 | 10 | 9 | AR\ | ARV | 50 | 110 | 80 | 122 | 85 | 90 |
| N | 69 | 43 | 26 | 37 | 11 | 10 | Nor | ARV | 57 | 150 | 90 | 133 | 86 | 160 |
| S | 62 | 47 | 28 | 39 | 10 | 9 | Nor | Normal | 56 | 130 | 80 | 132 | 85 | 190 |

| | | | | | | | | | | | | | | |
|---|---|---|---|---|---|---|---|---|---|---|---|---|---|---|
| N | 61 | 46 | 29 | 35 | 10 | 10 | AR\ | ARV | 77 | 150 | 100 | 124 | 86 | 150 |
| S | 65 | 45 | 27 | 40 | 11 | 10 | ARV/IAO disc | | 79 | 164 | 89 | 141 | 90 | 216 |
| N | 66 | 55 | 32 | 42 | 13 | 13 | Imd | Imdiscret | 53 | 130 | 80 | 145 | 86 | 180 |
| N | 68 | 49 | 37 | 34 | 10 | 11 | AR\ | ARV | 79 | 110 | 70 | 140 | 85 | 140 |
| S | 53 | 56 | 43 | 39 | 12 | 8 | Pseudonorma | | 64 | 140 | 90 | 131 | 81 | 160 |
| S | 50 | 54 | 34 | 39 | 10 | 9 | ARV | | 60 | 140 | 70 | 147 | 94 | 150 |
| S | 66 | 52 | 33 | 41 | 9 | 9 | Nor | Normal | 62 | 130 | 40 | 93 | 64 | 140 |
| S | 66 | 49 | 30 | 39 | 10 | 9 | AR\ | ARV | 73 | 125 | 80 | 159 | 101 | 136 |
| N | 60 | 43 | 25 | 30 | 9 | 9 | AR\ | ARV | 64 | 160 | 80 | 121 | 75 | 160 |
| S | 48 | 52 | 41 | 37 | 11 | 8 | AR\ | ARV/Imd | 97 | 130 | 70 | 147 | 88 | 150 |
| N | 60 | 43 | 28 | 38 | 12 | 11 | ARV | | 78 | 140 | 80 | 135 | 91 | 150 |
| S | 69 | 41 | 27 | 39 | 10 | 10 | Pseudonorma | | 71 | 170 | 80 | 131 | 84 | 170 |
| N | 62 | 41 | 26 | 27 | 9 | 9 | AR\ | ARV | 61 | 110 | 80 | 143 | 86 | 90 |
| S | 70 | 59 | 34 | 41 | 12 | 11 | ARV | | 75 | 120 | 100 | 148 | 89 | 179 |
| S | 64 | 41 | 27 | 30 | 8 | 9 | Nor | Normal | 60 | 150 | 90 | 160 | 96 | 160 |
| N | 64 | 45 | 27 | 37 | 9 | 9 | AR\ | ARV | 51 | 110 | 70 | 146 | 89 | 150 |
| S | 65 | 46 | 30 | 41 | 12 | 11 | ARV | | 59 | 125 | 80 | 141 | 87 | 132 |
| S | 64 | 44 | 27 | 37 | 9 | 9 | ARV | | 77 | 140 | 90 | 145 | 95 | 130 |
| N | 66 | 49 | 30 | 39 | 10 | 10 | AR\ | ARV | 79 | 135 | 78 | 157 | 94 | 171 |
| N | 68 | 46 | 27 | 36 | 10 | 9 | AR\ | ARV | 67 | 140 | 80 | 137 | 85 | 150 |
| S | 63 | 40 | 28 | 38 | 12 | 12 | ARV | | 51 | 110 | 70 | 145 | 86 | 100 |
| N | 71 | 45 | 26 | 40 | 10 | 9 | ARV | | 75 | 120 | 60 | 138 | 90 | 120 |
| S | 70 | 52 | 30 | 41 | 10 | 9 | Pseudonorma | | 58 | 175 | 69 | 150 | 101 | 198 |
| S | 50 | 57 | 33 | 41 | 10 | 8 | ARV/hipoc inf | | 56 | 140 | 90 | 154 | 97 | 140 |
| S | 67 | 46 | 28 | 35 | 10 | 10 | AR\ | ARV | 78 | 130 | 90 | 139 | 88 | 170 |
| S | 50 | 55 | 37 | 34 | 9 | 8 | ARV/Ac medi | | 67 | 100 | 68 | 148 | 85 | 152 |
| S | 69 | 48 | 31 | 44 | 11 | 11 | ARV/EAO dis | | 52 | 130 | 70 | 142 | 92 | 160 |
| N | 66 | 40 | 25 | 40 | 13 | 12 | ARV/IAOdiscr | | 62 | 120 | 80 | 158 | 100 | 140 |
| S | 68 | 53 | 37 | 39 | 10 | 9 | ARV | | 45 | 160 | 70 | 152 | 96 | 180 |
| S | 59 | 54 | 37 | 43 | 9 | 9 | AR\ | ARV/Hipo | 52 | 132 | 91 | 132 | 78 | 140 |
| S | 69 | 45 | 28 | 36 | 13 | 11 | ARV | | 59 | 150 | 100 | 157 | 93 | 180 |
| S | 68 | 50 | 32 | 40 | 12 | 12 | ARV | | | | | | | |
| S | 65 | 45 | 29 | 34 | 9 | 9 | AR\ | ARV | 62 | 120 | 70 | 145 | 93 | 190 |
| N | 66 | 47 | 30 | 36 | 11 | 10 | Nor | Normal | 63 | 90 | 60 | 150 | 88 | 110 |
| S | 50 | 64 | 41 | 48 | 11 | 11 | IM discreta | | 84 | 120 | 80 | 159 | 89 | 190 |
| N | 63 | 45 | 28 | 41 | 13 | 12 | AR\ | ARV | 75 | 180 | 80 | 133 | 86 | 180 |
| N | 65 | 47 | 29 | 37 | 11 | 9 | Nor | Normal | 65 | 127 | 82 | 144 | 83 | 170 |
| S | 70 | 47 | 30 | 40 | 10 | 10 | Pseudonorma | | 50 | 120 | 72 | 152 | 100 | 180 |
| N | 55 | 54 | 40 | 45 | 10 | 10 | IMd | IMdiscret | 47 | 120 | 80 | 114 | 71 | 140 |
| S | 70 | 44 | 26 | 32 | 10 | 10 | normal | | 62 | 130 | 80 | 154 | 86 | 170 |
| N | 65 | 50 | 31 | 34 | 11 | 10 | Normal | | 56 | 114 | 66 | 154 | 89 | 160 |
| S | 62 | 48 | 29 | 29 | 8 | 8 | Nor | Normal | 65 | 130 | 80 | 137 | 87 | 150 |
| N | 70 | 47 | 30 | 35 | 11 | 11 | Nor | Normal | 84 | 155 | 95 | 156 | 93 | 175 |
| S | 65 | 41 | 27 | 40 | 10 | 10 | ARV | | 58 | 178 | 82 | 135 | 86 | 173 |
| S | 70 | 51 | 30 | 40 | 11 | 11 | AR\ | ARV | 77 | 138 | 76 | 158 | 93 | 136 |
| S | 62 | 54 | 36 | 39 | 9 | 9 | Nor | Normal | 58 | 120 | 70 | 137 | 86 | 150 |
| S | 66 | 50 | 30 | 38 | 11 | 10 | Normal | | 54 | 130 | 70 | 148 | 86 | 160 |
| N | 72 | 46 | 29 | 33 | 9 | 9 | Nor | Normal | 85 | 120 | 80 | 151 | 91 | 150 |
| S | 73 | 51 | 30 | 40 | 10 | 10 | Nor | Normal | 75 | 128 | 87 | 154 | 88 | 134 |
| S | 68 | 40 | 21 | 38 | 12 | 11 | AR\ | ARV | 70 | 111 | 73 | 144 | 85 | 110 |

| | | | | | | | | | | | | | |
|---|---|---|---|---|---|---|---|---|---|---|---|---|---|
| S | 60 | 47 | 30 | 39 | 12 | 11 | ARV/IAOdisc | 47 | 130 | 60 | 133 | 85 | 170 |
| N | 62 | 46 | 28 | 35 | 10 | 10 | Imd Imdiscret | 46 | 100 | 60 | 130 | 91 | 150 |
| S | 55 | 59 | 40 | 58 | 14 | 11 | IM moderada | 50 | 150 | 100 | 140 | 92 | 190 |
| S | 65 | 47 | 30 | 40 | 12 | 12 | ARV | 90 | 170 | 80 | 160 | 100 | 180 |
| N | 65 | 44 | 29 | 37 | 12 | 13 | ARV | 68 | 150 | 80 | 148 | 91 | 190 |
| N | 66 | 47 | 30 | 36 | 8 | 9 | Normal | 63 | 120 | 70 | 153 | 89 | 188 |
| S | 71 | 48 | 30 | 40 | 11 | 11 | ARV | 59 | 175 | 71 | 127 | 85 | 190 |
| S | 71 | 52 | 30 | 36 | 9 | 8 | ARV | 64 | 129 | 55 | 129 | 85 | 155 |
| S | 68 | 40 | 25 | 30 | 9 | 9 | Nor Normal | 55 | 90 | 80 | 143 | 87 | 170 |
| S | 61 | 49 | 30 | 35 | 11 | 10 | ARV | 56 | 130 | 70 | 103 | 65 | 140 |
| N | 62 | 48 | 27 | 35 | 10 | 9 | IMd IMdiscret | 77 | 140 | 60 | 139 | 87 | 150 |
| S | 68 | 45 | 28 | 40 | 9 | 9 | ARV ARV | 74 | 130 | 80 | 152 | 87 | 200 |
| N | 64 | 42 | 25 | 28 | 9 | 9 | ARV ARV | 88 | 140 | 70 | 142 | 90 | 190 |
| S | 60 | 51 | 34 | 34 | 9 | 9 | ARV/Ac basa | 88 | 140 | 70 | 142 | 90 | 190 |
| S | 57 | 48 | 31 | 38 | 11 | 11 | ARV ARV/hipc | 62 | 144 | 57 | 148 | 91 | 120 |
| S | 64 | 44 | 25 | 32 | 9 | 9 | ARV ARV | 83 | 120 | 70 | 164 | 98 | 140 |
| S | 60 | 47 | 30 | 49 | 11 | 10 | Pseudonorma | 53 | 178 | 83 | 126 | 87 | 195 |
| S | 55 | 58 | 39 | 40 | 9 | 8 | Pseudonorma | 47 | 121 | 48 | 142 | 88 | 134 |
| S | 66 | 41 | 26 | 33 | 10 | 9 | ARV/Imdiscre | 65 | 120 | 60 | 150 | 101 | 140 |
| N | 63 | 46 | 31 | 41 | 10 | 10 | ARV | 65 | 140 | 90 | 137 | 84 | 170 |
| S | 62 | 39 | 26 | 31 | 8 | 8 | Nor Normal | | | | | | |
| S | 68 | 42 | 28 | 40 | 9 | 9 | ARV | 90 | 150 | 70 | 164 | 100 | 170 |
| S | 50 | 49 | 31 | 40 | 12 | 9 | ARV/Hipoc in | 87 | 180 | 100 | 147 | 94 | 190 |
| S | 62 | 46 | 27 | 32 | 8 | 8 | Nor Normal | 52 | 90 | 60 | 144 | 81 | 130 |
| S | 67 | 48 | 31 | 35 | 10 | 10 | ARV | 52 | 138 | 70 | 143 | 88 | 156 |
| S | 72 | 45 | 27 | 41 | 11 | 11 | ARV ARV | 75 | 136 | 83 | 142 | 86 | 160 |
| S | 74 | 43 | 28 | 38 | 11 | 9 | Normal | | | | | | |
| S | 68 | 39 | 24 | 30 | 10 | 8 | IMdiscreta | | | | | | |
| S | 69 | 50 | 29 | 38 | 10 | 10 | Normal | 62 | 120 | 70 | 135 | 85 | 130 |
| S | 65 | 56 | 37 | 45 | 10 | 10 | ARV/Imdiscre | 64 | 160 | 90 | 132 | 91 | 180 |
| S | 60 | 42 | 26 | 31 | 9 | 9 | ARV | 84 | 110 | 70 | 146 | 93 | 129 |
| S | 62 | 45 | 29 | 37 | 9 | 9 | ARV/Hipoc ap | 60 | 160 | 80 | 98 | 60 | 140 |
| S | 65 | 47 | 30 | 35 | 9 | 9 | ARV | 63 | 130 | 80 | 154 | 99 | 130 |
| S | 69 | 36 | 24 | 28 | 8 | 9 | ARV | | | | | | |
| S | 65 | 39 | 27 | 34 | 9 | 7 | Normal | | | | | | |
| N | 60 | 50 | 34 | 35 | 12 | 11 | ARV/IAOdisc | 56 | 110 | 70 | 137 | 92 | 150 |
| N | 61 | 47 | 32 | 34 | 8 | 8 | ARV/Ac apica | 60 | 100 | 70 | 112 | 67 | 100 |
| S | 63 | 52 | 30 | 33 | 11 | 11 | ARV/Hipocine | 67 | 151 | 88 | 137 | 88 | 162 |
| S | 60 | 46 | 29 | 35 | 12 | 11 | ARV ARV/Acir | 69 | 120 | 90 | 157 | 93 | 140 |
| N | 68 | 40 | 26 | 38 | 12 | 11 | ARV/Imdiscre | 62 | 176 | 92 | 141 | 94 | 160 |
| S | 63 | 41 | 28 | 38 | 9 | 9 | ARV ARV | 70 | 170 | 90 | 144 | 90 | 130 |
| S | 65 | 46 | 29 | 29 | 8 | 8 | Normal | | | | | | |
| S | 55 | 61 | 42 | 41 | 11 | 11 | ARV/Imdisc/I | 43 | 113 | 65 | 144 | 88 | 129 |
| S | 64 | 52 | 35 | 40 | 10 | 10 | ARV/Hipoc ap | 55 | 120 | 70 | 152 | 92 | 130 |
| S | 62 | 53 | 36 | 44 | 10 | 9 | Normal | 54 | 124 | 72 | 163 | 100 | 170 |
| N | 60 | 44 | 28 | 43 | 17 | 15 | ARV | 57 | 114 | 76 | 147 | 90 | 149 |
| N | 63 | 48 | 28 | 36 | 9 | 9 | nor Normal | | | | | | |
| N | 66 | 52 | 31 | 36 | 10 | 10 | normal | 46 | 130 | 80 | 154 | 100 | 160 |
| N | 62 | 42 | 25 | 32 | 9 | 9 | IAO IAO/IM di | 65 | 160 | 80 | 154 | 100 | 150 |
| S | 47 | 60 | 46 | 38 | 12 | 8 | ARV/Hipo infe | 76 | 140 | 90 | 158 | 98 | 160 |

| | | | | | | | | | | | | | | |
|---|---|---|---|---|---|---|---|---|---|---|---|---|---|---|
| N | 63 | 45 | 27 | 34 | 8 | 8 | Normal | | 61 | 120 | 80 | 159 | 98 | 150 |
| S | 65 | 59 | 34 | 38 | 10 | 9 | Normal | | 85 | 120 | 80 | 159 | 94 | 180 |
| S | 62 | 50 | 39 | 44 | 10 | 8 | Normal | | | | | | | |
| S | 63 | 47 | 29 | 43 | 12 | 12 | AR\ | ARV/IM c | 56 | 160 | 70 | 135 | 92 | 100 |
| N | 64 | 46 | 26 | 32 | 9 | 9 | Nor | Normal | 59 | 130 | 70 | 138 | 88 | 150 |
| S | 74 | 42 | 22 | 42 | 10 | 9 | AR\ | ARV | 69 | 140 | 80 | 138 | 86 | 130 |
| S | 74 | 44 | 28 | 35 | 10 | 10 | Imdiscreta/IA | | | | | | | |
| S | 68 | 51 | 30 | 42 | 13 | 13 | ARV | | 66 | 140 | 90 | 156 | 100 | 170 |
| S | 70 | 44 | 28 | 35 | 9 | 9 | Normal | | | | | | | |
| S | 62 | 50 | 29 | 45 | 11 | 10 | Normal | | | | | | | |
| S | 50 | 60 | 40 | 44 | 13 | 12 | Pseudonorma | | 53 | 172 | 99 | 141 | 89 | 160 |
| S | 64 | 44 | 28 | 32 | 9 | 9 | AR\ | ARV | 61 | 120 | 70 | 159 | 94 | 130 |
| N | 67 | 43 | 27 | 41 | 10 | 10 | Normal | | 58 | 120 | 70 | 146 | 85 | 140 |
| S | 65 | 48 | 30 | 35 | 10 | 10 | AR\ | ARV | 60 | 150 | 70 | 160 | 91 | 180 |
| N | 70 | 45 | 28 | 33 | 8 | 8 | IAO discreta | | 61 | 140 | 80 | 153 | 96 | 150 |
| S | 62 | 43 | 28 | 33 | 9 | 9 | ARV | | 60 | 120 | 80 | 139 | 86 | 140 |
| S | 65 | 48 | 31 | 36 | 11 | 10 | AR\ | ARV | 56 | 130 | 70 | 140 | 89 | 180 |
| S | 68 | 50 | 31 | 40 | 11 | 11 | ARV/discreta | | 60 | 142 | 72 | 139 | 90 | 210 |
| S | 65 | 40 | 26 | 30 | 8 | 8 | AR\ | ARV/IM c | 56 | 120 | 70 | 155 | 97 | 150 |

| PAD p | Dose Dob (mcg | Atropina (mg) | Complicaç | Dor prec | Dor abd | ESV | HAS | Hipot | Nausea | Cefaleia |
|---|---|---|---|---|---|---|---|---|---|---|
| 90 | | | N | N | N | N | N | N | N | N |
| 78 | | | S | S | N | S | N | N | N | N |
| 90 | | | S | S | N | N | N | N | N | N |
| 75 | | | N | N | N | N | N | N | N | N |
| 80 | | | S | S | N | S | N | N | N | N |
| 60 | | | N | N | N | N | N | N | N | N |
| 80 | | | N | N | N | N | N | N | N | N |
| 93 | 40 | 2 | N | N | N | N | N | N | N | N |
| 74 | | | S | N | N | S | N | N | N | N |
| 99 | | | N | N | N | N | N | N | N | N |
| 70 | 20 | 0,25 | S | N | S | N | N | N | N | N |
| 110 | 20 | 0,5 | S | N | N | N | S | N | N | N |
| 110 | | | S | S | N | N | N | N | N | N |
| 80 | 40 | 1,5 | S | N | N | S | N | N | N | N |
| 70 | 40 | 1,5 | S | S | N | S | N | N | N | N |
| 90 | | | N | N | N | N | N | N | N | N |
| 60 | | | N | N | N | N | N | N | N | N |
| 80 | | | N | N | N | N | N | N | N | N |
| 76 | 40 | 2 | S | N | N | S | N | N | N | N |
| 70 | 20 | 0,25 | S | S | N | N | N | N | N | N |
| 70 | 30 | 0,5 | S | N | N | S | N | N | N | N |
| 100 | 40 | 1,75 | S | N | N | S | N | N | N | N |
| 80 | | | S | N | N | S | N | N | N | N |
| 70 | 20 | 0,5 | N | N | N | N | N | N | N | N |
| 70 | | | N | N | N | N | N | N | N | N |
| 103 | 40 | 0 | N | N | N | N | N | N | N | N |
| 90 | 30 | 0,5 | S | N | N | S | N | N | N | N |
| 89 | 40 | 2 | N | N | N | N | N | N | N | N |
| 80 | 20 | 0,25 | S | S | N | N | N | N | N | N |
| 61 | 40 | 1,25 | N | N | N | N | N | N | N | N |
| 97 | | | N | N | N | N | N | N | N | N |
| 91 | | | S | S | N | N | N | N | N | N |
| 83 | | | N | N | N | N | N | N | N | N |
| 116 | 20 | 0 | S | N | N | S | S | N | N | N |
| 90 | | | S | N | N | S | N | N | N | N |
| 119 | | | S | N | N | N | S | N | N | N |
| 70 | 40 | 1,75 | S | N | N | S | N | N | N | N |
| 60 | | | S | N | N | S | N | N | N | N |
| 60 | 40 | 1 | S | S | N | S | N | N | N | N |
| 60 | 30 | 0,75 | S | N | N | S | N | N | N | N |
| 60 | | | S | N | N | N | N | S | N | N |
| 80 | | | S | N | N | S | N | N | N | N |
| 70 | 40 | 2 | N | N | N | N | N | N | N | N |
| 68 | 30 | 0,75 | S | S | N | S | N | N | N | N |
| | 40 | 1,5 | | | | | | | | |
| 77 | 30 | 0,5 | N | N | N | N | N | N | N | N |
| 50 | | | N | N | N | N | N | N | N | N |
| 80 | | | S | N | N | S | N | N | N | N |
| 60 | | | N | N | N | N | N | N | N | N |

| | | | | | | | | | | |
|---|---|---|---|---|---|---|---|---|---|---|
| 70 | | | S | N | N | S | N | N | N | N |
| 100 | 30 | 0 | N | N | N | N | N | N | N | N |
| 90 | | | N | N | N | N | N | N | N | N |
| 70 | | | S | S | N | S | N | N | N | N |
| 90 | 40 | 0 | N | N | N | N | N | N | N | N |
| 80 | 40 | 1,25 | N | N | N | N | N | N | N | N |
| 30 | | | S | S | N | S | N | N | N | N |
| 64 | | | S | N | N | S | N | N | N | N |
| 90 | | | N | N | N | N | N | N | N | N |
| 70 | | | S | S | | N | N | N | N | N |
| 72 | 20 | 0,75 | S | N | N | S | N | N | N | N |
| 90 | 40 | 0 | S | S | N | S | N | N | N | N |
| 60 | | | N | N | N | N | N | N | N | N |
| 103 | 30 | 0 | N | N | N | N | N | N | N | N |
| 90 | | | S | S | N | N | N | N | N | N |
| 80 | | | S | N | N | S | N | N | N | N |
| 66 | 30 | 1,25 | N | N | N | N | N | N | N | N |
| 80 | 20 | 0,5 | S | N | N | S | N | N | N | N |
| 82 | | | N | N | N | N | N | N | N | N |
| 80 | | | S | N | N | S | N | N | N | N |
| 70 | 30 | 1,5 | S | N | N | S | N | N | N | N |
| 60 | 20 | 0,25 | S | N | N | S | N | N | N | N |
| 68 | 20 | 0,5 | S | S | N | S | N | N | N | N |
| 90 | 30 | 1 | S | S | N | N | N | N | N | N |
| 90 | | | N | N | N | N | N | N | N | N |
| 96 | 40 | 2 | S | N | N | S | N | N | N | N |
| 80 | 30 | 1 | S | N | N | S | N | N | N | N |
| 100 | 30 | 0,5 | N | N | N | N | N | N | N | N |
| 100 | 30 | 0,75 | S | N | N | N | S | N | N | N |
| 70 | | | S | N | N | S | N | N | N | N |
| 80 | 30 | 0,5 | S | N | N | S | N | N | N | N |
| | 40 | 1,5 | | | | | | | | |
| 100 | | | S | N | N | N | N | S | N | N |
| 70 | | | S | S | N | S | N | S | N | N |
| 140 | 30 | 0,75 | S | N | N | S | S | N | N | N |
| 90 | | | N | N | N | N | N | N | N | N |
| 90 | | | N | N | N | N | N | N | N | N |
| 102 | 30 | 1,5 | S | S | N | N | S | N | N | N |
| 60 | | | S | S | N | N | N | N | N | N |
| 90 | 30 | 0,75 | N | N | N | N | N | N | N | N |
| 80 | 40 | 1 | N | N | N | N | N | N | N | N |
| 60 | | | S | N | N | S | N | N | N | N |
| 98 | | | N | N | N | N | N | N | N | N |
| 93 | 40 | 1,5 | S | S | N | N | N | N | N | N |
| 68 | | | S | S | N | N | N | N | N | N |
| 60 | | | S | N | N | S | N | N | N | N |
| 70 | 30 | 0,75 | S | N | N | S | N | N | N | N |
| 70 | | | S | N | N | S | N | N | N | N |
| 72 | | | S | S | N | S | N | N | N | S |
| 70 | | | S | S | N | N | N | N | N | N |

| | | | | | | | | | | |
|---|---|---|---|---|---|---|---|---|---|---|
| 80 | 40 | 2 | S | N | N | S | N | N | N | N |
| 80 | | | N | N | N | N | N | N | N | N |
| 140 | 30 | 0,75 | S | N | N | S | S | N | N | N |
| 60 | 30 | 0,25 | N | N | N | N | N | N | N | N |
| 110 | 40 | 1,5 | S | S | N | S | N | N | N | N |
| 56 | 30 | 1 | S | N | N | S | N | N | N | N |
| 52 | 40 | 2 | S | N | N | S | N | N | N | N |
| 52 | 40 | 2 | N | N | N | N | N | N | N | N |
| 100 | | | N | N | N | N | N | N | N | N |
| 80 | 40 | 0 | N | N | N | N | N | N | N | N |
| 50 | | | S | S | N | N | N | N | N | N |
| 111 | | | S | N | N | N | S | N | N | N |
| 80 | | | S | N | N | N | N | N | S | N |
| 80 | | | S | N | N | N | N | N | S | N |
| 104 | | | S | S | N | S | N | N | N | N |
| 90 | | | S | N | N | S | N | N | N | N |
| 94 | 40 | 0 | N | N | N | N | N | N | N | N |
| 41 | 40 | 1,25 | N | N | N | N | N | N | N | N |
| 60 | 40 | 1,25 | N | N | N | N | N | N | N | N |
| 100 | 30 | 0,25 | S | S | N | N | N | N | N | N |
| | | | N | N | N | N | N | N | N | N |
| 50 | 30 | 0,5 | S | N | N | S | N | N | N | N |
| 100 | 20 | 0,5 | N | N | N | N | N | N | N | N |
| 70 | | | S | N | N | N | N | N | N | N |
| 76 | 30 | 1,5 | N | N | N | N | N | N | N | N |
| 70 | | | N | N | N | N | N | N | N | N |
| | | | N | N | N | N | N | N | N | N |
| | | | N | N | N | N | N | N | N | N |
| 90 | 40 | 2 | N | N | N | N | N | N | N | N |
| 90 | 30 | 0 | S | N | N | S | N | N | N | N |
| 72 | 20 | 0,5 | S | S | N | N | N | N | N | N |
| 80 | 30 | 0,5 | N | N | N | N | N | N | N | N |
| 80 | 20 | 0,5 | N | N | N | N | N | N | N | N |
| | | | N | N | N | N | N | N | N | N |
| | | | N | N | N | N | N | N | N | N |
| 80 | | | N | N | N | N | N | N | N | N |
| 60 | 40 | 2 | N | N | N | N | N | N | N | N |
| 64 | 30 | 1,25 | S | N | N | S | N | N | N | N |
| 90 | | | S | N | N | S | N | N | N | N |
| 92 | 20 | 0,75 | S | N | N | S | N | N | N | N |
| 70 | | | S | N | N | S | N | N | N | N |
| | | | N | N | N | N | N | N | N | N |
| 79 | 40 | 2 | S | S | N | S | N | N | N | N |
| 80 | 40 | 2 | S | N | N | S | N | N | N | N |
| 78 | 40 | 1,75 | N | N | N | N | N | N | N | N |
| 73 | 30 | 0,75 | S | N | N | S | N | N | N | N |
| | | | N | N | N | N | N | N | N | N |
| 90 | 40 | 1,25 | N | N | N | N | N | N | N | N |
| 80 | | | S | N | N | S | N | N | N | N |
| 80 | 30 | 1 | S | S | N | N | N | N | N | N |

| | | | | | | | | | | |
|---|---|---|---|---|---|---|---|---|---|---|
| 70 | 30 | 0,5 | S | N | N | S | N | N | N | N |
| 90 | 30 | 1 | N | N | N | N | N | N | N | N |
| | | | N | N | N | N | N | N | N | N |
| 70 | | | S | S | N | N | N | N | N | N |
| 70 | | | N | N | N | N | N | N | N | N |
| 80 | | | S | N | N | S | N | N | N | N |
| | | | N | N | N | N | N | N | N | N |
| 100 | 30 | 1 | S | S | N | N | N | N | N | N |
| | | | N | N | N | N | N | N | N | N |
| | | | N | N | N | N | N | N | N | N |
| 93 | 30 | 1 | S | S | N | S | N | N | N | N |
| 70 | | | N | N | N | N | N | N | N | N |
| 60 | | | N | N | N | N | N | N | N | N |
| 100 | | | S | N | N | N | S | N | N | N |
| 80 | 30 | 0,5 | N | N | N | N | N | N | N | N |
| 80 | 30 | 0,75 | S | S | N | S | N | N | N | N |
| 80 | | | N | N | N | N | N | N | N | N |
| 81 | 30 | | N | N | N | N | N | N | N | N |
| 90 | | | S | S | N | S | N | N | N | N |

| Tremor | Taquipneia | Tipo | CSD ANORMAL | QLD ANORMAL | Reserva B -DA | Reserva AxB - DA |
|---|---|---|---|---|---|---|
| N | N | 0 | S | S | 1,03 | 1,33 |
| N | N | Dor pr | S | S | 2,49 | 2,40 |
| N | N | Dor pr | N | N | 2,98 | 3,82 |
| N | N | 0 | S | S | 1,70 | 2,96 |
| N | N | Dor pr | N | N | 1,64 | 1,85 |
| N | N | 0 | N | N | 3,20 | 4,06 |
| N | N | 0 | N | N | 2,98 | 5,88 |
| N | N | 0 | N | N | 1,99 | 1,64 |
| N | N | ESV | N | N | 3,12 | 3,70 |
| N | N | 0 | N | N | 3,51 | 3,13 |
| N | N | Dor lo | N | N | 3,47 | 2,98 |
| N | N | HAS | S | S | 1,69 | 1,22 |
| N | N | Dor pr | S | S | 1,55 | 2,15 |
| N | N | ESV | N | N | 3,28 | 4,11 |
| N | N | Dor pr | N | N | 2,44 | 1,97 |
| N | N | 0 | N | N | 3,42 | 4,48 |
| N | N | 0 | N | N | 2,46 | 3,13 |
| N | N | 0 | N | N | 2,95 | 3,54 |
| N | N | ESV | N | N | 3,51 | 3,45 |
| N | N | Dor pr | N | N | 3,3 | 3,8 |
| N | N | ESV | N | N | 2,43 | 3,39 |
| N | N | ESV | S | S | 3,09 | 4,93 |
| N | N | ESV | N | S | 2,75 | 2,15 |
| N | N | 0 | N | S | 4,08 | 4,09 |
| N | N | 0 | N | S | 2,15 | 2,09 |
| N | N | 0 | N | N | 3,51 | 3,12 |
| N | N | ESV | S | S | 1,64 | 2,86 |
| N | N | 0 | S | S | 3,53 | 4,05 |
| N | N | Dor pr | S | S | 1,82 | 1,36 |
| N | N | 0 | N | N | 3,38 | 4,22 |
| N | N | 0 | N | N | 3,96 | 3,96 |
| N | N | Dor pr | S | S | 1,85 | 1,04 |
| N | N | 0 | N | N | 2,56 | 4,78 |
| N | N | Hipote | N | N | 4,2 | 4,49 |
| N | N | ESV | S | S | 2,44 | 3,12 |
| N | N | HAS | N | N | 2,69 | 2,46 |
| N | N | ESV | N | N | 3,8 | 2,92 |
| N | N | ESV | N | N | 2,75 | 2,30 |
| N | N | Dor pr | S | S | 1,6 | 2,66 |
| N | N | ESV | N | N | 2,46 | 1,65 |
| N | N | Hipote | S | S | 1,17 | 1,31 |
| N | N | ESV | N | N | 4,37 | 5,76 |
| N | N | 0 | S | S | 1,75 | 0,72 |
| N | N | Dor pr | N | S | 3,46 | 1,43 |
| | | | N | N | 3,17 | 4,84 |
| N | N | 0 | N | N | 3,5 | 3,89 |
| N | N | 0 | N | N | 2,57 | 3,24 |
| N | N | ESV | N | N | 3,61 | 4,80 |
| N | N | 0 | N | N | 4,15 | 6,03 |

| | | | | | | |
|---|---|---|---|---|---|---|
| N | N | ESV | S | S | 0,96 | 1,23 |
| N | N | 0 | N | N | 2,87 | 3,05 |
| N | N | 0 | N | N | 2,77 | 4,45 |
| N | N | Dor pr | N | N | 1,87 | 2,24 |
| N | N | 0 | N | S | 3,38 | 1,74 |
| N | N | 0 | S | S | 1,77 | 1,6 |
| N | N | Dor pr | S | S | 0,74 | 0,80 |
| N | N | ESV | N | S | 1,73 | 2,13 |
| N | N | 0 | N | N | 3,21 | 4,00 |
| N | N | Dor pr | S | S | 1,63 | 1,59 |
| N | N | ESV | N | N | 3,62 | 2,82 |
| N | N | Dor pr | N | S | 2,94 | 2,31 |
| N | N | 0 | S | S | 2,17 | 2,39 |
| N | N | 0 | N | N | 4,26 | 4,21 |
| N | N | Dor pr | N | N | 3,61 | 4,08 |
| N | N | ESV | N | N | 2,12 | 2,68 |
| N | N | 0 | N | N | 3,4 | 3,37 |
| N | N | ESV | N | S | 2,36 | 2,41 |
| N | N | 0 | N | N | 3,01 | 3,33 |
| N | N | ESV | N | N | 2,17 | 2,62 |
| N | N | ESV | N | N | 2,89 | 3,22 |
| N | N | ESV | S | S | 3,64 | 5,75 |
| N | N | Dor pr | N | N | 3,49 | 5,28 |
| N | N | Dor pr | S | S | 1,38 | 1,52 |
| N | N | 0 | N | N | 2,46 | 3,00 |
| N | N | ESV | S | S | 1,95 | 1,24 |
| N | N | ESV | N | N | 1,77 | 1,35 |
| N | N | 0 | N | S | 3,23 | 4,51 |
| N | N | HAS | N | N | 2,97 | 5,91 |
| N | N | ESV | S | S | 1,79 | 1,13 |
| N | N | ESV | N | N | 2,68 | 1,99 |
| | | | N | N | 3,54 | 4,27 |
| N | N | Hipote | S | S | 2,73 | 2,95 |
| N | N | Dor pr | S | S | 1,15 | 1,35 |
| N | N | HAS + | N | N | 2,77 | 2,04 |
| N | N | 0 | N | S | 1,54 | 2,50 |
| N | N | 0 | N | N | 3,15 | 3,07 |
| N | N | HAS + | N | N | 2,17 | 1,07 |
| N | N | Dor pr | S | S | 1,26 | 1,40 |
| N | N | 0 | S | S | 1,68 | 0,9 |
| N | N | 0 | N | N | 2,99 | 4,42 |
| N | N | ESV, I | N | N | 2,89 | 3,21 |
| N | N | 0 | N | N | 2,83 | 2,76 |
| N | N | Dor pr | N | S | 4,31 | 2,93 |
| N | N | Dor pr | S | S | 1,51 | 1,43 |
| N | N | ESV | N | N | 2,31 | 2,23 |
| N | N | ESV | N | N | 2,93 | 2,66 |
| N | N | ESV | N | N | 2,93 | 3,63 |
| N | N | Dor pr | N | N | 3,04 | 5,20 |
| N | N | Dor pr | S | S | 1,78 | 1,96 |

| | | | | | | |
|---|---|---|---|---|---|---|
| N | N | ESV | N | N | 2,84 | 2,5 |
| N | N | 0 | N | N | 1,27 | 1,84 |
| N | N | HAS + | S | S | 1,85 | 2,29 |
| N | N | 0 | S | S | 1,93 | 1,84 |
| N | N | Dor pr | S | S | 1,85 | 0,87 |
| N | N | ESV | N | N | 2,49 | 2,81 |
| N | N | ESV | N | N | 3,51 | 2,97 |
| N | N | 0 | N | N | 2,78 | 2,29 |
| N | N | 0 | N | N | 2,98 | 4,36 |
| N | N | 0 | N | N | 4,09 | 5,52 |
| N | N | Dor pr | S | S | 2,76 | 3,63 |
| N | N | HAS | S | S | 3,38 | 2,91 |
| N | S | Taquip | S | S | 2,95 | 4,47 |
| N | S | Taquip | N | N | 3,63 | 4,5 |
| N | N | Dor pr | S | S | 1,95 | 1,84 |
| N | N | ESV | N | N | 2,79 | 4,38 |
| N | N | 0 | N | N | 4,29 | 4,52 |
| N | N | 0 | N | N | 3,8 | 2,06 |
| N | N | 0 | N | N | 3,74 | 2,48 |
| N | N | Dor pr | N | N | 2,84 | 2,99 |
| N | N | 0 | N | N | 3,21 | 3,79 |
| N | N | ESV | S | S | 1,75 | 2,13 |
| N | N | 0 | S | S | 1,46 | 1,41 |
| S | N | Tremo | N | N | 2,47 | 3,66 |
| N | N | 0 | N | N | 3,02 | 3,7 |
| N | N | 0 | N | S | 2,72 | 3,29 |
| N | N | 0 | N | S | 3,87 | 4,25 |
| N | N | 0 | S | S | 3,57 | 4,65 |
| N | N | 0 | N | N | 3,65 | 2,13 |
| N | N | ESV | S | S | 3,32 | 3,11 |
| N | N | Dor pr | S | S | 1,51 | 1,15 |
| N | N | 0 | S | S | 1,35 | 2,91 |
| N | N | 0 | N | N | 3,68 | 3,59 |
| N | N | 0 | N | N | 3,62 | 5,27 |
| N | N | 0 | S | S | 1,09 | 1,57 |
| N | N | 0 | N | N | 1,93 | 2,60 |
| N | N | 0 | S | S | 3,12 | 2,48 |
| N | N | ESV | S | S | 1,69 | 2,74 |
| N | N | ESV | S | S | 2,55 | 3,09 |
| N | N | ESV | N | N | 3,83 | 5,32 |
| N | N | ESV | N | N | 3,06 | 2,86 |
| N | N | 0 | N | N | 2,19 | 3,11 |
| N | N | Dor pr | S | S | 1,7 | 1,97 |
| N | N | ESV | N | S | 3,31 | 3,1 |
| N | N | 0 | N | N | 3,42 | 5,16 |
| N | N | ESV | N | N | 3,68 | 3,87 |
| N | N | 0 | S | S | 1,96 | 1,61 |
| N | N | 0 | N | N | 3,8 | 6,58 |
| N | N | ESV | N | N | 0,70 | 0,97 |
| N | N | Dor pr | N | S | 1,53 | 1,59 |

| | | | | | | |
|---|---|---|---|---|---|---|
| N | N | ESV | N | N | 4,2 | 3,47 |
| N | N | 0 | N | N | 3,75 | 3,25 |
| N | N | 0 | S | S | 1,16 | 1,74 |
| N | N | Dor pr | S | S | 1,67 | 2,04 |
| N | N | 0 | N | N | 2,27 | 2,59 |
| N | N | ESV | S | S | 1,66 | 2,03 |
| N | N | 0 | N | N | 3,64 | 5,36 |
| N | N | Dor pr | S | S | 1,54 | 1,18 |
| N | N | 0 | S | S | 1,38 | 2,25 |
| N | N | 0 | S | S | 2,11 | 2,87 |
| N | N | Dor pr | S | S | 1,99 | 1,5 |
| N | N | 0 | N | N | 3,90 | 4,63 |
| N | N | 0 | N | N | 4,27 | 2,81 |
| N | N | HAS | S | S | 1,64 | 1,82 |
| N | N | 0 | N | N | 3,81 | 2,19 |
| N | N | Dor pr | N | N | 4,15 | 4,16 |
| N | N | 0 | N | N | 2,56 | 2,41 |
| N | N | 0 | N | N | 1,68 | 3,84 |
| N | N | Dor pr | N | N | 2,35 | 4,58 |

| Reserva B -CX | Reserva AxB - CX | Reserva B -CD | Reserva AxB - CD | IAM | RM | ATC | AI | Morte Total |
|---|---|---|---|---|---|---|---|---|
| 0,99 | 1,16 | 0,99 | 1,13 | N | N | N | N | S |
| 1,73 | 2,25 | 2,24 | 2,59 | N | N | N | S | N |
| 3,96 | 4,68 | 3,04 | 4,68 | | | | | |
| 1,15 | 1,30 | 1,46 | 1,39 | | | | | |
| 2,96 | 3,24 | 2,26 | 2,57 | N | N | S | N | N |
| 3,25 | 3,77 | 2,33 | 3,13 | | | | | |
| 3,25 | 4,43 | 3,05 | 2,96 | | | | | |
| 3,64 | 3,95 | 2,10 | 1,83 | | | | | |
| 2,63 | 3,15 | 3,08 | 4,00 | | | | | |
| 3,82 | 4,58 | 2,98 | 2,51 | | | | | |
| 3,25 | 3,01 | 3,57 | 3,43 | | | | | |
| 1,64 | 1,97 | 2,44 | 1,59 | N | S | N | S | N |
| 0,90 | 1,04 | 1,02 | 1,17 | | | | | |
| 3,44 | 2,01 | 2,7 | 2,95 | | | | | |
| 1,86 | 1,6 | 1,42 | 0,81 | N | S | N | N | N |
| 2,42 | 2,91 | 2,77 | 3,43 | | | | | |
| 2,81 | 3,63 | 7,72 | 10,33 | | | | | |
| 3,41 | 6,10 | 3,29 | 3,87 | | | | | |
| 3,33 | 2,6 | 3,1 | 3,26 | S | N | N | N | N |
| 3,47 | 2,48 | 3,63 | 4,97 | | | | | |
| 2,64 | 3,01 | 2,47 | 2,61 | | | | | |
| 2,6 | 0,75 | 1,98 | 0,87 | | | | | |
| 1,48 | 2,13 | 1,70 | 1,25 | N | S | N | N | N |
| 3,31 | 3,45 | 3,61 | 2,81 | | | | | |
| 1,42 | 2,67 | 1,50 | 2,44 | N | S | N | N | N |
| 3,73 | 4,06 | 3,63 | 3,47 | | | | | |
| 1,93 | 1,68 | 1,87 | 1,44 | | | | | |
| 1,98 | 1,62 | 1,87 | 1,5 | | | | | |
| 1,33 | 1,4 | 1,3 | 1,01 | N | S | N | N | N |
| 3,02 | 3,23 | 3,08 | 2,57 | | | | | |
| 4,89 | 5,84 | 3,95 | 4,93 | | | | | |
| 2,00 | 0,84 | 1,26 | 2,36 | N | S | N | N | N |
| 2,53 | 3,12 | 2,98 | 6,04 | | | | | |
| 3,27 | 5,83 | 3,31 | 5,24 | | | | | |
| 1,15 | 1,49 | 2,71 | 3,19 | | | | | |
| 3,38 | 6,04 | 1,69 | 1,11 | | | | | |
| 2,86 | 3,29 | 3,76 | 4,25 | | | | | |
| 2,93 | 1,41 | 3,76 | 6,81 | | | | | |
| 1,72 | 1,29 | 2,36 | 1,43 | N | N | N | N | S |
| 3,22 | 2 | 3,05 | 3,4 | | | | | |
| 1,01 | 1,46 | 1,17 | 1,27 | | | | | |
| 4,34 | 6,63 | 3,84 | 5,42 | | | | | |
| 1,82 | 1,56 | 1,48 | 1,32 | | | | | |
| 3,37 | 3,98 | 1,65 | 0,62 | | | | | |
| 3,27 | 5,72 | 3,55 | 3,54 | | | | | |
| 2,84 | 2,88 | 3,4 | 3,48 | | | | | |
| 3,73 | 3,70 | 1,67 | 2,11 | | | | | |
| 3,12 | 4,13 | 3,04 | 3,85 | | | | | |
| 4,13 | 5,89 | 4,69 | 5,82 | | | | | |

| | | | | | | | | |
|---|---|---|---|---|---|---|---|---|
| 2,15 | 2,75 | 2,09 | 2,57 | | | | | |
| 2,76 | 2,24 | 2,61 | 3,36 | | | | | |
| 4,33 | 6,07 | 2,74 | 4,09 | | | | | |
| 1,71 | 2,09 | 1,92 | 2,73 | | | | | |
| 4,34 | 3,07 | 1,8 | 1,98 | | | | | |
| 1,74 | 2,96 | 1,79 | 1,59 | | | | | |
| 1,20 | 1,66 | 1,41 | 1,77 | N | S | N | N | N |
| 1,69 | 2,25 | 2,14 | 2,42 | N | S | N | S | N |
| 2,55 | 3,10 | 2,09 | 2,81 | | | | | |
| 1,48 | 1,40 | 1,76 | 1,52 | N | S | N | S | N |
| 4,27 | 3,7 | 3,36 | 4,05 | | | | | |
| 1,75 | 2,19 | 1,94 | 0,8 | N | S | N | N | N |
| 2,31 | 2,49 | 1,82 | 1,92 | | | | | |
| 5,37 | 5,21 | 3,23 | 4,18 | | | | | |
| 2,86 | 3,81 | 2,17 | 2,35 | | | | | |
| 2,27 | 2,82 | 7,11 | 8,05 | | | | | |
| 3,54 | 2,96 | 3,29 | 3,52 | | | | | |
| 1,83 | 1,38 | 3,27 | 3,1 | N | N | S | N | N |
| 2,74 | 4,94 | 3,48 | 4,47 | | | | | |
| 2,31 | 2,67 | 1,82 | 2,28 | N | S | N | S | N |
| 3,65 | 4,19 | 2,72 | 3,92 | | | | | |
| 1,96 | 3,16 | 2,48 | 2,2 | | | | | |
| 2,86 | 3,44 | 2,79 | 3,76 | | | | | |
| 1,25 | 1,14 | 1,33 | 1,91 | N | S | N | N | N |
| 2,81 | 3,52 | 3,22 | 3,88 | | | | | |
| 2,61 | 4,79 | 3,24 | 3,82 | | | | | |
| 2,78 | 2,03 | 2,69 | 2,45 | | | | | |
| 3,13 | 2,87 | 4,29 | 5,93 | | | | | |
| 4,75 | 6,77 | 2,77 | 2,64 | | | | | |
| 1,76 | 2,39 | 2,61 | 1,70 | N | S | N | N | N |
| 2,47 | 2,56 | 3,02 | 2,9 | | | | | |
| 4,75 | 4,59 | 3,86 | 2,39 | | | | | |
| 1,88 | 2,35 | 2,23 | 2,76 | N | S | N | N | N |
| 0,63 | 0,79 | 1,57 | 1,59 | N | S | N | N | N |
| 2,98 | 2,46 | 2,91 | 2,05 | | | | | |
| 1,61 | 2,06 | 1,56 | 1,52 | S | N | N | N | N |
| 3,69 | 3,84 | 3,09 | 1,90 | | | | | |
| 1,76 | 1,1 | 2,62 | 2,57 | | | | | |
| 0,84 | 0,99 | 2,01 | 2,97 | | | | | |
| 2,93 | 1,02 | 3,28 | 2,93 | | | | | |
| 3,62 | 2,86 | 3,26 | 2,39 | | | | | |
| 1,80 | 1,72 | 2,31 | 2,54 | | | | | |
| 3,14 | 2,57 | 3,28 | 3,87 | | | | | |
| 4,2 | 4,83 | 1,88 | 1,12 | N | S | N | N | N |
| 1,34 | 1,13 | 1,34 | 1,21 | | | | | |
| 2,53 | 1,77 | 3,00 | 2,83 | | | | | |
| 3,2 | 5,33 | 3,33 | 3,19 | | | | | |
| 3,58 | 4,50 | 1,74 | 2,78 | | | | | |
| 2,16 | 5,04 | 2,96 | 2,60 | | | | | |
| 2,10 | 2,30 | 2,36 | 2,14 | N | S | N | N | N |

| | | | | | | | | |
|---|---|---|---|---|---|---|---|---|
| 3,1 | 1,93 | 3,7 | 3,84 | | | | | |
| 3,12 | 3,74 | 0,92 | 1,39 | S | N | N | N | S |
| 2,45 | 2,11 | 2,4 | 2,13 | | | | | |
| 1,66 | 1,32 | 3,75 | 4,56 | | | | | |
| 1,94 | 2,17 | 2,04 | 1,94 | | | | | |
| 3,32 | 2,05 | 2,68 | 3,46 | | | | | |
| 3,19 | 3,38 | 3,75 | 3,92 | | | | | |
| 3,3 | 1,7 | 2,61 | 3,25 | | | | | |
| 2,33 | 2,45 | 3,36 | 4,05 | N | N | S | S | N |
| 1,8 | 3,41 | 1,84 | 1,79 | N | S | N | N | N |
| 3,44 | 5,49 | 2,59 | 3,41 | N | N | S | N | N |
| 2,93 | 2,63 | 2,93 | 1,93 | | | | | |
| 2,41 | 3,10 | 2,72 | 3,84 | | | | | |
| 4,44 | 5,56 | 3,16 | 4,75 | | | | | |
| 2,44 | 2,71 | 1,90 | 2,04 | N | N | N | S | N |
| 2,54 | 3,17 | 4,01 | 4,98 | | | | | |
| 4,13 | 4,14 | 4,1 | 3,88 | N | S | N | N | N |
| 3,75 | 3,19 | 3,35 | 3,31 | | | | | |
| 3,44 | 3,44 | 3,9 | 4,21 | | | | | |
| 2,8 | 2,1 | 2,97 | 2,14 | | | | | |
| 3,89 | 5,20 | 2,95 | 3,91 | | | | | |
| 2,87 | 1,66 | 3,06 | 2,33 | | | | | |
| 1,61 | 1,81 | 1,45 | 1,6 | | | | | |
| 3,15 | 3,70 | 0,77 | 0,89 | | | | | |
| 2,48 | 3,33 | 3,91 | 4,51 | | | | | |
| 2,55 | 3,66 | 3,00 | 2,14 | | | | | |
| 4,86 | 6,08 | 3,29 | 3,84 | | | | | |
| 2,46 | 2,92 | 2,51 | 3,18 | | | | | |
| 3,38 | 3,61 | 3,08 | 1,89 | | | | | |
| 1,89 | 1,65 | 1,88 | 1,85 | | | | | |
| 1,66 | 1,53 | 2,23 | 1,84 | | | | | |
| 1,5 | 2,34 | 1,74 | 1,72 | N | S | N | N | N |
| 3,88 | 4,35 | 3,65 | 4,04 | | | | | |
| 2,94 | 3,50 | 2,91 | 4,07 | | | | | |
| 1,56 | 2,06 | 0,62 | 0,69 | N | N | N | S | N |
| NA | NA | 3,06 | 4,88 | | | | | |
| 2,45 | 7,21 | 3,33 | 3,61 | | | | | |
| 1,73 | 2,22 | 1,88 | 2,67 | | | | | |
| 2,53 | 4,06 | 1,73 | 1,00 | | | | | |
| 3,27 | 6,12 | 2,98 | 2,76 | | | | | |
| 3,00 | 3,24 | 2,61 | 2,71 | | | | | |
| 1,87 | 2,19 | 1,99 | 2,41 | | | | | |
| 1,69 | 1,67 | 1,79 | 2,87 | | | | | |
| 4,19 | 6,25 | 3,33 | 4,21 | | | | | |
| 3,17 | 5,32 | 3,1 | 3,77 | | | | | |
| 2,97 | 3,13 | 3,04 | 4,84 | | | | | |
| 1,50 | 2,56 | 2,43 | 3,14 | N | S | N | N | N |
| 3,49 | 2,09 | 3,6 | 5,83 | | | | | |
| 0,54 | 0,73 | 1,56 | 2,08 | S | S | N | N | N |
| 2,17 | 3,29 | 2,89 | 2,28 | N | S | N | N | N |

| | | | | | | | | | |
|---|---|---|---|---|---|---|---|---|---|
| | 3,52 | 3,43 | 3,71 | 4,45 | | | | | |
| | 3,98 | 5,37 | 3,57 | 1,22 | | | | | |
| | 2,02 | 2,77 | 1,52 | 2,40 | N | S | N | S | N |
| | 2,89 | 1,65 | 2,29 | 3,02 | | | | | |
| | 2,36 | 2,93 | 1,91 | 2,38 | | | | | |
| | 1,49 | 2,25 | 1,66 | 1,64 | N | N | S | S | N |
| | 2,69 | 3,71 | 2,85 | 4,38 | | | | | |
| | 3,26 | 4,78 | 3,03 | 7,57 | | | | | |
| | 1,26 | 1,60 | 1,69 | 2,28 | N | S | N | S | N |
| | 1,93 | 3,11 | 1,29 | 1,83 | | | | | |
| | 1,8 | 2,45 | 3,71 | 2,36 | | | | | |
| NA | | NA | 2,04 | 2,51 | | | | | |
| | 4,05 | 5,62 | 3,21 | 3,27 | | | | | |
| | 2,98 | 2,12 | 1,70 | 2,35 | | | | | |
| | 2,39 | 3,11 | 3,48 | 3,71 | | | | | |
| | 3,59 | 4,78 | 3,47 | 3,73 | | | | | |
| | 2,64 | 2,54 | 2,84 | 4,57 | N | N | N | N | S |
| | 1,72 | 1,9 | 1,85 | 2,98 | | | | | |
| | 2,98 | 3,45 | 2,42 | 3,01 | | | | | |

| Causa | Morte cardiaca | TCE | DA | DG1 | DG2 | DLIS | CX | MG 1 | MG 2 | CD | DP | VP |
|---|---|---|---|---|---|---|---|---|---|---|---|---|
| ICC | S | 0 | 40 | 90 | 0 | 0 | 0 | 0 | 0 | 0 | 100 | 0 |
| SC | N | 0 | 0 | 0 | 0 | 0 | 100 | 100 | 0 | 0 | 0 | 0 |
| | | 0 | 0 | 0 | 0 | 0 | 0 | 0 | 0 | 0 | 0 | 0 |
| | | 0 | 71 | 0 | 0 | 0 | 95 | 0 | 95 | 88 | 0 | 0 |
| SC | N | 0 | 30 | 90 | 0 | 0 | 0 | 0 | 0 | 0 | 0 | 100 |
| | | 0 | 50 | 0 | 0 | 0 | 0 | 0 | 0 | 0 | 0 | 0 |
| | | 0 | 0 | 0 | 0 | 0 | 0 | 0 | 0 | 0 | 0 | 0 |
| | | 0 | 0 | 0 | 0 | 0 | 0 | 0 | 0 | 0 | 0 | 0 |
| | | 0 | 0 | 0 | 0 | 0 | 0 | 0 | 0 | 0 | 0 | 0 |
| | | 0 | 0 | 0 | 0 | 0 | 0 | 0 | 0 | 0 | 0 | 0 |
| | | 0 | 0 | 0 | 0 | 0 | 0 | 0 | 0 | 0 | 0 | 0 |
| SC | N | 0 | 100 | 0 | 0 | 0 | 0 | 80 | 70 | 0 | 0 | 0 |
| | | 30 | 100 | 60 | 0 | 0 | 0 | 0 | 0 | 0 | 0 | 0 |
| | | 0 | 0 | 0 | 0 | 0 | 0 | 0 | 0 | 54 | 0 | 0 |
| SC | N | 0 | 0 | 80 | 0 | 70 | 0 | 0 | 0 | 100 | 0 | 0 |
| | | 0 | 0 | 0 | 0 | 0 | 0 | 0 | 0 | 0 | 0 | 0 |
| | | 0 | 30 | 0 | 0 | 0 | 0 | 0 | 0 | 50 | 0 | 0 |
| | | 0 | 0 | 0 | 0 | 0 | 0 | 0 | 0 | 0 | 0 | 0 |
| SC | N | 0 | 0 | 0 | 0 | 0 | 0 | 0 | 0 | 0 | 0 | 0 |
| | | 0 | 0 | 0 | 0 | 0 | 0 | 0 | 0 | 0 | 0 | 0 |
| | | 0 | 0 | 0 | 0 | 0 | 0 | 0 | 0 | 0 | 0 | 0 |
| | | 0 | 31 | 0 | 0 | 0 | 76 | 90 | 0 | 90 | 0 | 0 |
| SC | N | 0 | 0 | 0 | 0 | 0 | 81 | 0 | 0 | 100 | 0 | 0 |
| | | 0 | 0 | 0 | 0 | 0 | 0 | 0 | 0 | 0 | 0 | 0 |
| SC | N | 0 | 0 | 0 | 0 | 0 | 83 | 62 | 0 | 59 | 0 | 0 |
| | | 0 | 0 | 0 | 0 | 0 | 0 | 0 | 0 | 59 | 0 | 0 |
| | | 0 | 100 | 100 | 100 | 0 | 100 | 100 | 100 | 100 | 0 | 0 |
| | | 0 | 0 | 0 | 0 | 0 | 0 | 0 | 0 | 100 | 0 | 0 |
| SC | N | 0 | 78 | 100 | 100 | 0 | 69 | 100 | 0 | 100 | 0 | 0 |
| | | 0 | 0 | 0 | 0 | 0 | 0 | 0 | 0 | 0 | 0 | 0 |
| | | 0 | 0 | 0 | 0 | 0 | 0 | 0 | 0 | 0 | 0 | 0 |
| SC | N | 0 | 100 | 0 | 0 | 0 | 96 | 0 | 86 | 82 | 59 | 70 |
| | | 0 | 0 | 0 | 0 | 0 | 0 | 0 | 0 | 0 | 0 | 0 |
| | | 0 | 0 | 0 | 0 | 0 | 0 | 0 | 0 | 0 | 0 | 0 |
| | | 0 | 0 | 0 | 0 | 0 | 0 | 0 | 0 | 0 | 0 | 0 |
| | | 0 | 0 | 0 | 0 | 0 | 0 | 0 | 0 | 0 | 0 | 0 |
| | | 0 | 0 | 0 | 0 | 0 | 0 | 0 | 0 | 0 | 0 | 0 |
| | | 0 | 0 | 43 | 0 | 0 | 0 | 0 | 0 | 0 | 0 | 0 |
| Arritmia | S | 0 | 57 | 51 | 0 | 0 | 0 | 72 | 0 | 78 | 61 | 0 |
| | | 0 | 0 | 0 | 0 | 0 | 0 | 0 | 0 | 0 | 0 | 0 |
| | | 0 | 70 | 70 | 60 | 0 | 0 | 0 | 0 | 90 | 0 | 0 |
| | | 0 | 0 | 0 | 0 | 0 | 0 | 0 | 0 | 0 | 0 | 0 |
| | | 0 | 0 | 0 | 0 | 0 | 0 | 100 | 0 | 0 | 100 | 0 |
| | | 0 | 0 | 0 | 0 | 0 | 0 | 0 | 0 | 100 | 0 | 0 |
| | | 0 | 0 | 0 | 0 | 0 | 0 | 0 | 0 | 80 | 0 | 100 |
| | | 0 | 0 | 0 | 0 | 0 | 0 | 0 | 0 | 0 | 0 | 0 |
| | | 0 | 0 | 50 | 0 | 0 | 40 | 0 | 0 | 0 | 0 | 0 |
| | | 0 | 0 | 0 | 0 | 0 | 0 | 0 | 0 | 0 | 0 | 0 |
| | | 0 | 0 | 0 | 0 | 0 | 0 | 0 | 0 | 0 | 0 | 0 |

| | | | | | | | | | | | | |
|---|---|---|---|---|---|---|---|---|---|---|---|---|
| | | 0 | 70 | 0 | 70 | 0 | 0 | 0 | 0 | 0 | 0 | 0 |
| | | 0 | 0 | 70 | 33 | 0 | 60 | 0 | 0 | 55 | 0 | 0 |
| | | 0 | 0 | 0 | 0 | 0 | 0 | 0 | 0 | 0 | 0 | 0 |
| | | 0 | 0 | 0 | 0 | 0 | 0 | 0 | 0 | 0 | 0 | 0 |
| | | 0 | 0 | 0 | 0 | 0 | 0 | 0 | 0 | 100 | 100 | 100 |
| | | 0 | 0 | 64 | 51 | 0 | 100 | 0 | 100 | 0 | 0 | 0 |
| SC | N | 0 | 90 | 70 | 0 | 0 | 80 | 90 | 70 | 100 | 0 | 0 |
| SC | N | 0 | 100 | 0 | 0 | 0 | 53 | 0 | 0 | 59 | 0 | 0 |
| | | 0 | 0 | 0 | 0 | 0 | 0 | 50 | 0 | 0 | 0 | 0 |
| SC | N | 50 | 0 | 50 | 60 | 0 | 0 | 80 | 0 | 100 | 0 | 0 |
| | | 0 | 53 | 0 | 0 | 0 | 0 | 0 | 0 | 0 | 0 | 0 |
| SC | N | 0 | 43 | 0 | 0 | 0 | 0 | 70 | 0 | 44 | 0 | 0 |
| | | 0 | 0 | 0 | 0 | 0 | 0 | 0 | 0 | 0 | 0 | 0 |
| | | 0 | 0 | 0 | 0 | 0 | 0 | 0 | 0 | 0 | 0 | 0 |
| | | 0 | 0 | 0 | 0 | 0 | 0 | 0 | 0 | 0 | 0 | 0 |
| | | 0 | 0 | 0 | 0 | 0 | 0 | 0 | 0 | 0 | 0 | 0 |
| | | 0 | 0 | 0 | 0 | 0 | 0 | 0 | 0 | 0 | 0 | 0 |
| SC | N | 0 | 41 | 0 | 0 | 0 | 100 | 0 | 0 | 0 | 0 | 0 |
| | | 0 | 0 | 0 | 0 | 0 | 0 | 0 | 0 | 0 | 0 | 0 |
| SC | N | 0 | 0 | 0 | 0 | 0 | 0 | 0 | 0 | 0 | 0 | 0 |
| | | 0 | 0 | 49 | 0 | 0 | 0 | 0 | 0 | 0 | 0 | 0 |
| | | 0 | 0 | 71 | 0 | 0 | 0 | 0 | 0 | 0 | 0 | 0 |
| | | 0 | 0 | 0 | 0 | 0 | 0 | 0 | 0 | 0 | 0 | 0 |
| SC | N | 0 | 54 | 0 | 0 | 0 | 64 | 0 | 0 | 71 | 0 | 0 |
| | | 0 | 0 | 0 | 0 | 0 | 0 | 0 | 0 | 0 | 0 | 0 |
| | | 0 | 0 | 0 | 0 | 0 | 0 | 0 | 0 | 0 | 0 | 0 |
| | | 0 | 58 | 0 | 0 | 0 | 62 | 0 | 0 | 0 | 0 | 0 |
| | | 0 | 0 | 0 | 0 | 0 | 0 | 0 | 0 | 0 | 0 | 0 |
| | | 0 | 54 | 0 | 0 | 0 | 0 | 0 | 0 | 0 | 0 | 0 |
| SC | N | 0 | 71 | 0 | 0 | 0 | 52 | 0 | 90 | 100 | 0 | 0 |
| | | 0 | 54 | 0 | 61 | 0 | 0 | 0 | 0 | 0 | 0 | 0 |
| | | 31 | 0 | 0 | 0 | 0 | 0 | 0 | 0 | 100 | 0 | 0 |
| SC | N | 0 | 50 | 90 | 0 | 0 | 70 | 0 | 0 | 40 | 0 | 0 |
| SC | N | 0 | 90 | 0 | 0 | 0 | 40 | 0 | 0 | 70 | 0 | 0 |
| | | 0 | 0 | 0 | 0 | 0 | 0 | 0 | 0 | 0 | 0 | 0 |
| SC | N | 0 | 50 | 0 | 0 | 0 | 48 | 0 | 0 | 67 | 100 | 100 |
| | | 0 | 0 | 0 | 0 | 0 | 0 | 0 | 0 | 0 | 0 | 0 |
| | | 35 | 67 | 0 | 0 | 0 | 100 | 0 | 0 | 0 | 0 | 0 |
| | | 0 | 0 | 70 | 0 | 0 | 80 | 40 | 70 | 90 | 0 | 0 |
| | | 0 | 0 | 0 | 0 | 0 | 0 | 0 | 0 | 0 | 0 | 0 |
| | | 0 | 0 | 0 | 0 | 0 | 0 | 0 | 0 | 0 | 0 | 0 |
| | | 0 | 50 | 0 | 0 | 0 | 71 | 0 | 0 | 44 | 0 | 83 |
| | | 0 | 0 | 0 | 0 | 0 | 0 | 0 | 0 | 0 | 0 | 0 |
| SC | N | 0 | 71 | 0 | 0 | 0 | 52 | 0 | 0 | 100 | 0 | 0 |
| | | 0 | 0 | 0 | 0 | 0 | 0 | 0 | 0 | 0 | 0 | 0 |
| | | 0 | 0 | 0 | 0 | 0 | 0 | 0 | 0 | 0 | 0 | 0 |
| | | 0 | 0 | 0 | 0 | 0 | 0 | 0 | 0 | 0 | 0 | 0 |
| | | 0 | 0 | 0 | 0 | 0 | 0 | 0 | 0 | 0 | 0 | 0 |
| | | 0 | 0 | 0 | 0 | 0 | 0 | 0 | 0 | 0 | 0 | 0 |
| SC | N | 0 | 100 | 0 | 0 | 0 | 0 | 0 | 0 | 0 | 0 | 0 |

| | | | | | | | | | | | | |
|---|---|---|---|---|---|---|---|---|---|---|---|---|
| | | 0 | 0 | 0 | 0 | 0 | 0 | 0 | 0 | 0 | 0 | 0 |
| IAM | S | 0 | 90 | 70 | 0 | 0 | 60 | 100 | 0 | 0 | 0 | 0 |
| | | 0 | 46 | 58 | 0 | 0 | 0 | 0 | 0 | 0 | 0 | 0 |
| | | 0 | 0 | 0 | 0 | 0 | 0 | 0 | 66 | 62 | 62 | 71 |
| | | 0 | 69 | 59 | 0 | 73 | 0 | 76 | 0 | 0 | 75 | 0 |
| | | 0 | 0 | 0 | 0 | 0 | 0 | 0 | 0 | 0 | 0 | 0 |
| | | 0 | 0 | 42 | 0 | 0 | 0 | 0 | 0 | 0 | 0 | 0 |
| | | 0 | 0 | 0 | 0 | 0 | 0 | 0 | 0 | 0 | 0 | 0 |
| SC | N | 0 | 0 | 0 | 0 | 0 | 0 | 0 | 80 | 0 | 0 | 0 |
| SC | N | 0 | 69 | 50 | 0 | 0 | 100 | 57 | 0 | 0 | 0 | 0 |
| SC | N | 0 | 70 | 0 | 0 | 0 | 0 | 0 | 0 | 100 | 0 | 0 |
| | | 0 | 0 | 0 | 0 | 0 | 0 | 0 | 0 | 0 | 0 | 0 |
| | | 0 | 0 | 0 | 0 | 0 | 0 | 0 | 0 | 0 | 0 | 0 |
| | | 0 | 0 | 0 | 0 | 0 | 0 | 0 | 0 | 0 | 0 | 0 |
| SC | N | 0 | 100 | 50 | 0 | 0 | 52 | 85 | 0 | 100 | 0 | 0 |
| | | 0 | 0 | 0 | 0 | 0 | 0 | 0 | 0 | 0 | 0 | 0 |
| SC | N | 62 | 62 | 0 | 0 | 0 | 0 | 0 | 63 | 30 | 0 | 0 |
| | | 0 | 0 | 0 | 53 | 0 | 0 | 0 | 0 | 32 | 0 | 0 |
| | | 0 | 30 | 0 | 0 | 0 | 0 | 0 | 0 | 55 | 0 | 0 |
| | | 0 | 0 | 0 | 0 | 0 | 0 | 0 | 0 | 0 | 0 | 0 |
| | | 0 | 0 | 0 | 0 | 0 | 0 | 0 | 0 | 0 | 0 | 0 |
| | | 0 | 30 | 0 | 0 | 0 | 0 | 0 | 0 | 0 | 0 | 0 |
| | | 0 | 52 | 0 | 52 | 0 | 100 | 88 | 0 | 33 | 0 | 0 |
| | | 0 | 0 | 0 | 0 | 0 | 0 | 0 | 0 | 0 | 0 | 0 |
| | | 0 | 0 | 0 | 0 | 0 | 0 | 0 | 0 | 0 | 0 | 0 |
| | | 0 | 0 | 0 | 0 | 0 | 0 | 0 | 0 | 0 | 0 | 0 |
| | | 0 | 100 | 40 | 0 | 0 | 0 | 0 | 0 | 0 | 0 | 40 |
| | | 0 | 0 | 0 | 0 | 0 | 0 | 0 | 0 | 0 | 0 | 0 |
| | | 0 | 0 | 0 | 0 | 0 | 0 | 0 | 0 | 0 | 0 | 0 |
| | | 0 | 66 | 65 | 0 | 89 | 56 | 0 | 88 | 0 | 57 | 0 |
| | | 0 | 100 | 0 | 0 | 0 | 0 | 0 | 0 | 0 | 0 | 0 |
| SC | N | 0 | 65 | 70 | 0 | 0 | 100 | 68 | 0 | 62 | 52 | 52 |
| | | 0 | 0 | 0 | 0 | 0 | 0 | 0 | 0 | 0 | 0 | 0 |
| | | 0 | 60 | 90 | 0 | 0 | 0 | 40 | 0 | 40 | 0 | 0 |
| SC | N | 0 | 60 | 0 | 0 | 0 | 0 | 90 | 0 | 0 | 0 | 0 |
| | | 0 | 0 | 70 | 80 | 0 | 0 | 0 | 0 | 0 | 60 | 0 |
| | | 0 | 0 | 0 | 0 | 0 | 0 | 0 | 0 | 0 | 0 | 0 |
| | | 0 | 100 | 0 | 0 | 0 | 81 | 0 | 0 | 72 | 69 | 0 |
| | | 0 | 0 | 0 | 0 | 0 | 0 | 0 | 56 | 100 | 0 | 0 |
| | | 0 | 0 | 0 | 0 | 0 | 0 | 0 | 0 | 0 | 0 | 0 |
| | | 0 | 0 | 0 | 0 | 0 | 0 | 0 | 0 | 0 | 0 | 0 |
| | | 0 | 0 | 0 | 0 | 0 | 0 | 0 | 0 | 0 | 0 | 0 |
| | | 55 | 83 | 0 | 0 | 0 | 0 | 0 | 0 | 67 | 71 | 0 |
| | | 0 | 39 | 74 | 0 | 0 | 59 | 0 | 0 | 68 | 0 | 0 |
| | | 0 | 0 | 0 | 0 | 0 | 0 | 0 | 0 | 0 | 0 | 0 |
| | | 0 | 0 | 0 | 0 | 0 | 46 | 0 | 100 | 53 | 31 | 41 |
| SC | N | 0 | 70 | 0 | 0 | 0 | 77 | 0 | 0 | 100 | 0 | 0 |
| | | 0 | 0 | 0 | 0 | 0 | 0 | 0 | 0 | 0 | 0 | 0 |
| SC | N | 0 | 0 | 0 | 0 | 0 | 0 | 0 | 0 | 80 | 0 | 0 |
| SC | N | 0 | 67 | 66 | 56 | 0 | 61 | 0 | 0 | 39 | 0 | 0 |

| | | | | | | | | | | | | |
|---|---|---|---|---|---|---|---|---|---|---|---|---|
| | | 0 | 0 | 0 | 0 | 0 | 0 | 0 | 0 | 0 | 0 | 0 |
| | | 0 | 0 | 0 | 0 | 0 | 0 | 0 | 0 | 0 | 0 | 0 |
| SC | N | 0 | 70 | 0 | 0 | 50 | 80 | 0 | 70 | 100 | 0 | 0 |
| | | 0 | 73 | 0 | 0 | 0 | 63 | 40 | 0 | 100 | 0 | 0 |
| | | 0 | 0 | 0 | 0 | 0 | 0 | 0 | 0 | 0 | 0 | 0 |
| SC | N | 0 | 81 | 78 | 0 | 0 | 0 | 0 | 0 | 0 | 0 | 0 |
| | | 0 | 0 | 70 | 0 | 0 | 0 | 0 | 50 | 0 | 0 | 0 |
| | | 0 | 100 | 0 | 0 | 0 | 0 | 0 | 0 | 0 | 0 | 0 |
| SC | N | 60 | 70 | 0 | 0 | 0 | 100 | 100 | 0 | 0 | 0 | 0 |
| | | 0 | 80 | 90 | 0 | 0 | 0 | 0 | 0 | 70 | 0 | 0 |
| | | 0 | 65 | 0 | 70 | 0 | 100 | 0 | 0 | 0 | 84 | 0 |
| | | 0 | 0 | 0 | 0 | 0 | 0 | 0 | 0 | 0 | 0 | 0 |
| | | 0 | 0 | 0 | 0 | 0 | 0 | 0 | 0 | 0 | 0 | 0 |
| | | 0 | 0 | 0 | 0 | 0 | 0 | 0 | 0 | 0 | 0 | 0 |
| | | 0 | 0 | 0 | 0 | 0 | 0 | 0 | 0 | 0 | 0 | 0 |
| | | 0 | 0 | 0 | 0 | 0 | 0 | 0 | 0 | 0 | 0 | 0 |
| Adenocarci | N | 0 | 0 | 0 | 0 | 0 | 0 | 0 | 0 | 0 | 0 | 0 |
| | | 0 | 59 | 0 | 0 | 0 | 62 | 0 | 0 | 100 | 0 | 0 |
| | | 0 | 0 | 0 | 0 | 0 | 0 | 0 | 0 | 0 | 0 | 0 |
